# Exome Sequencing of 75 Individuals from Multiply Affected Coeliac Families and Large Scale Resequencing Follow Up

**DOI:** 10.1371/journal.pone.0116845

**Published:** 2015-01-30

**Authors:** Vanisha Mistry, Nicholas A. Bockett, Adam P. Levine, Muddassar M. Mirza, Karen A. Hunt, Paul J. Ciclitira, Holger Hummerich, Susan L. Neuhausen, Michael A. Simpson, Vincent Plagnol, David A. van Heel

**Affiliations:** 1 Blizard Institute, Barts and The London School of Medicine and Dentistry, 4 Newark Street, London E1 2AT, United Kingdom; 2 Division of Medicine, University College London, London, WC1E 6JF, United Kingdom; 3 UCL Advanced Diagnostics, Molecular Profiling Laboratory, Sarah Cannon-UCL Laboratories, Ground Floor, Shropshire House, 1 Capper Street, London, WC1E 6JA, United Kingdom; 4 King’s College London, Division of Diabetes and Nutritional Sciences, Gastroenterology, The Rayne Institute, St Thomas’ Hospital, Westminster Bridge Road, London SE1 7EH, United Kingdom; 5 Medical Research Council Prion Unit, Department of Neurodegenerative Disease, University College London Institute of Neurology, London WC1N 3BG, United Kingdom; 6 Department of Population Sciences, Beckman Research Institute of City of Hope, Duarte, California 91010, United States of America; 7 Division of Genetics and Molecular Medicine, Kings College London School of Medicine, 8^th^ Floor Tower Wing, Guy’s Hospital, London SE1 9RY, United Kingdom; 8 University College London Genetics Institute, Gower Street, London WC1E 6BT, United Kingdom; University of Southern California, UNITED STATES

## Abstract

Coeliac disease (CeD) is a highly heritable common autoimmune disease involving chronic small intestinal inflammation in response to dietary wheat. The human leukocyte antigen (HLA) region, and 40 newer regions identified by genome wide association studies (GWAS) and dense fine mapping, account for ∼40% of the disease heritability. We hypothesized that in pedigrees with multiple individuals with CeD rare [minor allele frequency (MAF) <0.5%] mutations of larger effect size (odds ratios of ∼ 2–5) might exist. We sequenced the exomes of 75 coeliac individuals of European ancestry from 55 multiply affected families. We selected interesting variants and genes for further follow up using a combination of: an assessment of shared variants between related subjects, a model-free linkage test, and gene burden tests for multiple, potentially causal, variants. We next performed highly multiplexed amplicon resequencing of all RefSeq exons from 24 candidate genes selected on the basis of the exome sequencing data in 2,248 unrelated coeliac cases and 2,230 controls. 1,335 variants with a 99.9% genotyping call rate were observed in 4,478 samples, of which 939 were present in coding regions of 24 genes (Ti/Tv 2.99). 91.7% of coding variants were rare (MAF <0.5%) and 60% were novel. Gene burden tests performed on rare functional variants identified no significant associations (p<1×10^−3^) in the resequenced candidate genes. Our strategy of sequencing multiply affected families with deep follow up of candidate genes has not identified any new CeD risk mutations.

## Introduction

Coeliac disease (CeD) is a common complex disease of the small intestine that occurs in approximately 1% of individuals of white European ancestry [1]. In susceptible people, ingestion of gluten peptides found in wheat, rye or barley results in a T-cell mediated immune response leading to villous atrophy, diarrhea and weight loss, amongst other symptoms. The role of HLA-DQ2 (particularly the HLA-DQ2.5 heterodimer encoded by the DQA1*0501 and DQB1*0201 alleles) and HLA-DQ8 in presenting negatively charged gluten peptides to gluten-specific T cells, eliciting an immune response, has been widely recognized [2,3,4,5]. Ninety seven and a half percent of people with CeD express/carry HLA-DQ2.5/DQ8 compared to only 47.5% of healthy people [6]. HLA DQ2.5/DQ8 is therefore not the sole component for disease risk. The current number of non-HLA genetic loci found through GWAS, and more recently dense fine mapping and resequencing studies, is 40 (58 independent signals) [6,7,8]. These studies have identified common and low frequency variation in CeD, with modest effect sizes that account for ∼13.7% of heritability (∼40% including HLA). In the last ten years, a vast number of discoveries identifying genetic variants strongly associated with susceptibility to complex traits have been made, with similar heritability estimates e.g. only 13.6% of total disease variance to Crohn’s disease risk is explained by 163 inflammatory bowel disease regions [9].

Since GWAS have typically not identified a large fraction of disease heritability (many of the loci found have individually small effects on disease phenotype) efforts have been directed toward discovering the contribution of rare variants to disease risk, according to the rare variant-common disease hypothesis [10]. Exome sequencing is a powerful tool for identifying rare variation in protein coding regions of the genome [11,12,13]. The method has been highly successful in detecting causal mutations in rare, Mendelian-type diseases [14,15,16] and has had some success in complex quantitative traits showing evidence of involvement of a few rare (1–5% allele frequency) and many ultra-rare/near-private mutations in disease genes [17,18,19]. Furthermore, the effects of deleted exons and premature stop codons can be easily explained in terms of impact on protein function, not only in rare disease but also for complex common disease without a clear mode of inheritance. For example, rare (MAF <3%) protective *IFIH1* mutations against type 1 diabetes (T1D) suggest a causative factor may be a host response to an enterovirus [20], whereas cytokine secretion by peripheral mononuclear cells has been recognised as being important in the pathogenesis of Crohn’s disease, in part through its defect in the context of *NOD2* mutations [21]. Although there is no current example of a high risk rare mutation in CeD, Crohn’s disease which has similar heritability provides examples: three major risk mutations in *NOD2* of population allele frequencies of ∼1–3% with homozygous genotypes conferring odds ratios of ∼15 for disease susceptibility under an additive model [22,23]. Other examples of rare coding variants in common disease are *ANGPTL4* in high-density lipoprotein cholesterol levels, *IFIHI* in T1D, *TREX1* in systemic lupus erythematosus, and *CARD14* in psoriasis [19,20,24,25].

Motivated by these collective findings, we designed a study to investigate whether we could identify rare mutations with high penetrance for CeD in pedigrees with a large number of CeD cases. Combined with multiple analytical strategies and a non-parametric linkage test, we identified 24 candidate genes for a follow-up deep resequencing study. To obtain an exhaustive picture of the genetic variation at these loci, rather than genotyping specific candidate variants, we resequenced all exons of these candidate genes in a large cohort of CeD cases and healthy controls.

## Results

**Figure 1:**
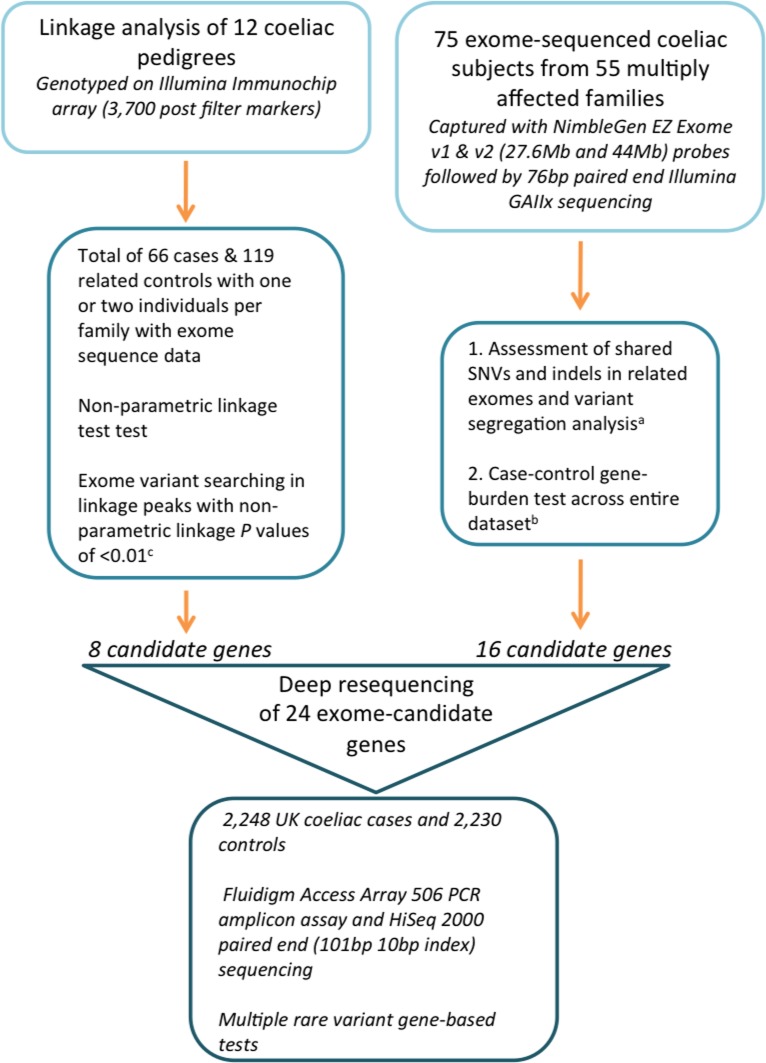
Analytical design of our study of rare variation in CeD. Only post quality filtered SNVs and indels were included in each analytical test. A, Not in dbSNP132, <5% MAF in 1000G, <10% MAF in coeliac exomes, not in 101 control exomes (54 ultra rare diseases from Kings College London and 47 Environmental Genome Project samples from University of Washington). B, Rare allele defined as MAF <0.5% in 1000G (n = 1092) for 220 controls and 41 unrelated coeliac exomes. C, MAF <0.5%, only variants predicted to be damaging and regions without duplications.

Our analytical strategy to locate highly penetrant rare variants clustering in coeliac pedigrees consisted of three components, outlined in Fig. 1. Firstly, we performed a discovery exome sequencing study in 75 coeliac individuals from 55 multiply affected CeD families (2 < number of affected persons per family < 14, S1 Table). All the families used in his study exhibited a dominant-like inheritance model and were at least two generations, with some extending to three or four generations. The families were selected from a worldwide set of collaborations. We first asked whether the number of cases in these families is consistent with what is known of the genetic architecture of CeD. This analysis is complicated by two factors. Firstly, our selection criteria create inherent biases: such families may not carry rare highly penetrant variants, and the excess of cases may be the result of chance alone. Secondly, while we gathered the most accurate pedigree information available, some unaffected individuals may be unreported. With these limitations in mind, we used the R-package Mangrove [26] to quantify whether the observed number of affected individuals is consistent with expectations based on known CeD prevalence and genetic architecture. We focused on the twelve families for which the pedigree is well described and examined 57 risk variants (S2 Table), including the HLA subtype inferred from ImmunoChip genotype, owing to its major impact on CeD risk. When examining all families we found the number of cases to greatly exceed expectations (76 affected compared to a 95% upper bound of 36, S1 Fig.). These results provide some support for the hypothesis that rare highly penetrant variants may be present in these families.

Two or more affected individuals per family were exome sequenced following the assumption that distantly related affected individuals would be enriched for segregating disease-causing single nucleotide variants (SNVs) and insertion-deletions (indels). Sequenced individuals were typically first cousins, first cousins once or twice removed, or grand relationships (between 3–12.5% genetic sharing). Our analytical methods included filtering for shared variants in related exomes, variant segregation tests in families and gene-burden tests across unrelated exomes (one exome per multiply affected CeD pedigree) and controls to detect an excess of rare variation in immune-pathway genes. Secondly, we selected a subset of pedigrees (twelve of the largest pedigrees out of the 55 in the entire dataset) to perform a linkage test using SNP markers from the Illumina ImmunoChip SNP array to define shared chromosomal intervals, thus restricting the search space for high-risk exonic variants in these families. From each pedigree, one or two individuals had exome-sequence data (these individuals were part of the 75 CeD sample set) and family-specific exonic variants within linkage intervals were prioritised. Our final follow-up study consisted of deep targeted resequencing of 24 candidate genes in a larger sample set including 2,248 coeliac subjects and 2,230 controls, to increase the power for rare variant identification.

### 1. Exome sequencing of 75 CeD individuals from multiply affected pedigrees

We performed exome capture and next generation sequencing of one to three (where available) key individuals from each coeliac pedigree (S1 Table). We obtained an average of 26,674,245 million reads per single exome, of which 22,700,447 were unique (S3 Table). One sequencing lane provided an optimal ∼50x mean read-depth (on-target, non-duplicate reads), sufficient to call on average 14,758 variants per exome (S3 Table and S2 Fig.). A total of 81,460 SNVs and 3,700 indels were observed in 75 exomes, and 38% (33,323) of these were novel. Of the 33,232 novel SNVs and indels, 5,839 were loss of function (LoF, defined as a mutation that causes reduced or complete loss of protein function).

For validation and specificity of variant calls, we compared genotypes from 26 Hap300 genotyped samples to 26 overlapping exome-sequenced samples: 99.96% of exome SNP calls were concordant with Hap300 genotype SNP calls from the same samples (total of 49,551 heterozygote calls in both datasets and 21 heterozygous calls in sequence but homozygous in array data). Within this set of call positions, the high concordance with array-based genotypes (for the Hap300 well-typed SNPs) provided an estimate of sensitivity for rare variant detection, as rare variants are largely expected to be heterozygous. For further detection of the specificity of our sequencing dataset, we additionally Sanger sequenced thirty-seven SNVs in the 75 individuals who had exome sequencing, of which three SNVs were false positive in the sequencing data (false positive rate = 8.1%).

#### 1.1 Shared exome variants between related subjects and segregation analysis

To identify novel and rare variants that were shared between related exomes the following filters were applied to SNVs and indels (Fig. 1): <5% MAF in 1000G (2011 data release), <10% MAF in coeliac exomes, and not present in 101 control exomes (54 extreme rare diseases from Kings College London and 47 Environmental Genome Project samples from the University of Washington). We used 1000G to define variant allele frequencies in our case dataset and set a MAF of <5% in 1000G in order to retain variants that were not common at a population-wide level. The control dataset contained 101 individuals and therefore did not provide a population-wide allele frequency, but was useful in filtering for novel variants in our dataset. Table 1 contains shared variants in exome-sequenced related subjects from six multiply affected families. Two or three subjects were sequenced per family and family relationships ranged from first cousins, first cousins once removed and grand relations. All variants were either nonsense or nonsynonymous missense SNVs. Effects on protein function were predicted by PolyPhen v2.2.2 [27] and SIFT v1.03 [28].

**Table 1:** Rare nonsynonymous single nucleotide variants located in immune genes and shared by related coeliac individuals in multiply affected families.

**Family ID**	**No. of exomes**	**Relationship**	**Selected SNVs of interest**	**Gene**	**PolyPhen prediction**	**In dbSNP132 or 1000G?**	**SIFT score**	**Cases validated / Cases tested**
FAM002	2	1^st^ cousins	c.617G>A	*C4PBA*	Benign	No	0.24	1/2
			c.58C>T	*TNFRSF13B*	Probably damaging	No	0	2/2
			c.517G>A	*TRAF4*	Benign	Yes	0.02	2/2
FAM006	2	1^st^ cousins once removed	c.66T>G	*RAF1*	Possibly damaging	No	0.03	2/2
			c.223G>A	*MAP4K2*	Possibly damaging	No	0	2/2
FAM007	2	1^st^ cousins	c.1251C>A	*CFTR*	Benign	dbSNP132	0.57	1/2
			c.1232T>C	*TNFRSF10A*	Probably damaging	1000G	0	2/2
			c.961C>T	*HAS1*	Possibly damaging	No	0.01	2/2
NEU4768	2	1^st^ cousins once removed	c.588A>C	*C1QBP*	Possibly damaging	No	0.11	2/2
BRK	2	Grand-uncle and grand nephew	c.184C>T*	*TNFRSF21*	Benign	No	0.3	2/2
NEU4801	3	1^st^ cousins, 1^st^ cousins twice removed and grand-nephew	c.70G>A*	*IL12R*	Probably damaging	No	0	3/3

SIFT scores range from 0 to 1, where < = 0.05 is predicted damaging and >0.05 is predicted tolerant. *Variants for segregation analysis.

**Figure 2:**
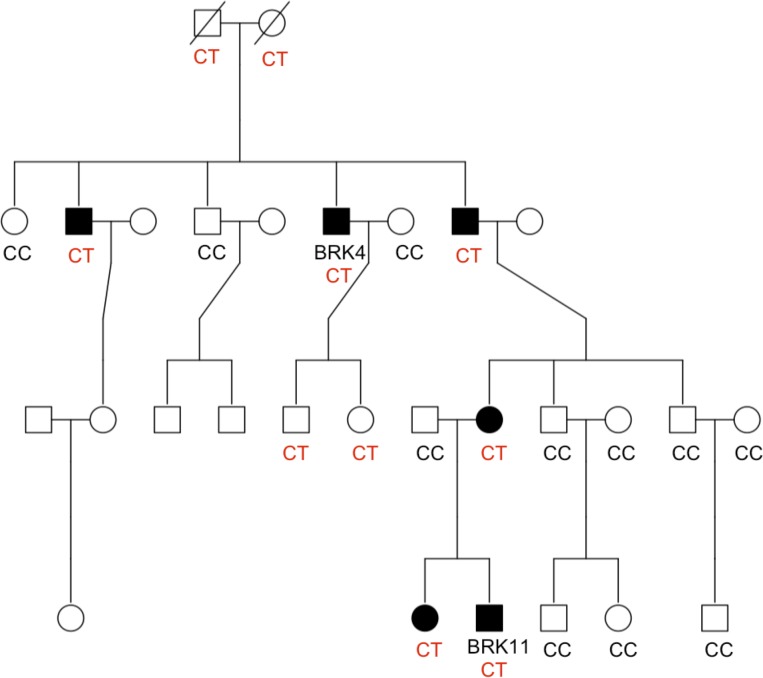
Segregation result for a novel c.184C>T (p.G62S) SNV in *TNFRSF21* in the BRK family. Variant c.184C>T (p.G62S) was sequenced in 20 individuals; DNA for five members was not available. BRK4 and BRK11 were selected for exome sequencing.

**Figure 3:**
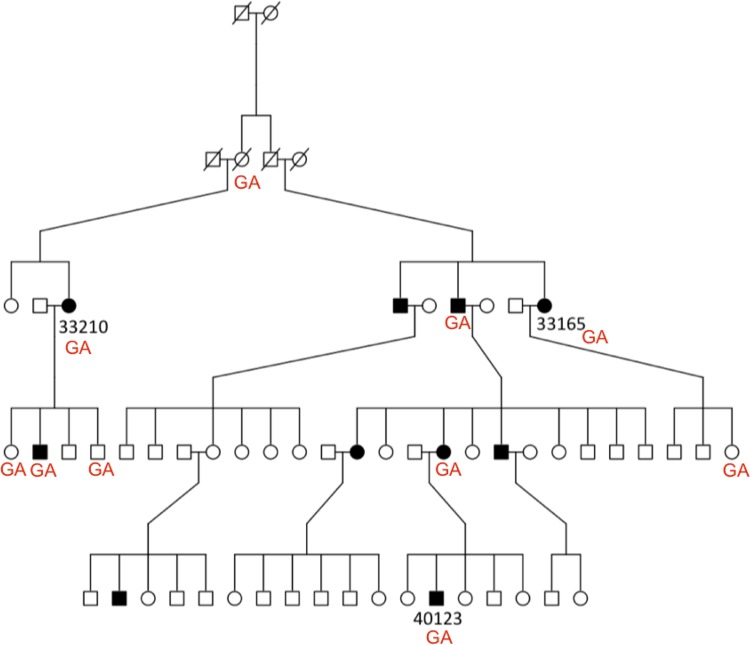
Segregation result for a novel c.70G>A (p.V24I) SNV in *IL21R* in the entire Neu4801 family. All other individuals carry homozygous GG wild type alleles (genotypes not shown on figure).

Tests for variant segregation were performed on families BRK and NEU4801, where DNA was available from additional relatives. Fig. 2 illustrates segregation results for a novel nonsynonymous missense substitution, c.184C>T (p.G62S), in *TNFRSF21* (NM_014452.3). Expression of this gene, also known as *DR6*, is down regulated in active T cells and DR6-deficient mice display reduced *CTLA4* expression, CD4^+^ T cell proliferation and T-helper cell differentiation implicating a possible role in inflammation [29,30]. The variant was validated by Sanger sequencing in all five coeliac cases and two of 13 unaffected relatives in the BRK family. However, we note that this follow-up segregation work only requires two meiosis to observe segregation between this variant and the remaining non-obligate CeD cases; hence, while consistent, this segregation result is not significant. Moreover, the presence of two heterozygous genotypes in unaffected individuals suggests that if the *TNFRSF21* c.184C>T (p.G62S) variant is implicated in CeD it cannot be fully penetrant. Fig. 3 illustrates segregation results for a nonsynonymous missense substitution, c.70G>A (p.V24I), in *IL21R* (NM_181079.4). This cytokine receptor for interleukin 21 is selectively expressed in lymphoid tissues and is important for proliferation and differentiation of B cells, T cells and NK cell expansion [31]. From Sanger sequencing, the variant was present in six of ten coeliac cases (three of whom were known to be heterozygous from the exome sequencing) and four of 37 unaffected relatives. The wild type genotype, GG, was observed in all other family members. This variant did not follow a segregation pattern with disease.

The remaining variants in Table 1 were validated by Sanger sequencing, except those in *CFTR* and *C4PBA*. We selected all the genes where these variants are located for follow-up deep amplicon resequencing: *TNFRSF13B, TRAF4, RAF1, MAP4K2, TNFRSF10A, HAS1, IL12RB* and *C1QPB* (Table 1 and S5 Table for specific immune functions of candidate genes and any known association or relevance to other autoimmune or gut diseases.).

#### 1.2 Single SNV and gene-burden tests on all exonic variants

**Figure 4:**
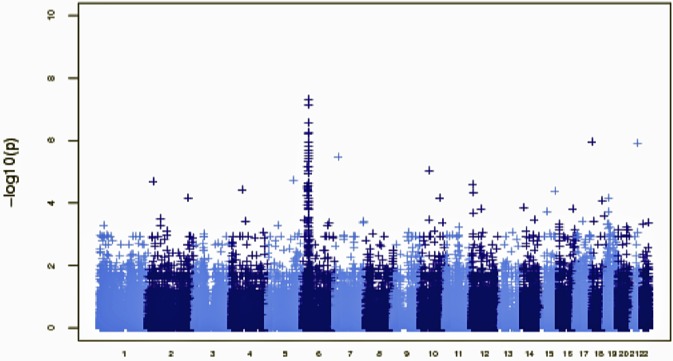
Manhattan plot of single-SNP tests comparing case data (n = 41, one case per multiply affected family) with 220 control samples.

A second approach to account for all identified sequenced variants was to perform single SNV and gene-level burden tests (Fig. 1). This analysis removed related individuals from the CeD exomes, leaving 41 unrelated CeD exomes and 220 neurological disorder control exomes. The single SNV test compared variant calls from cases and controls, similar to the test one would apply in a GWAS. We note that the power of this analysis is low owing to the small sample size (S5A Fig.). Nevertheless, an excess of rare variants in the HLA-complex on chromosome 6 was observed, with *p* values < 10^−7^ as illustrated in the Manhattan plot (Fig. 4). No other regions contained SNVs reaching *p*< = 10^−7^.

We then applied an aggregate test for rare variants by comparing numbers of variants within a gene to the genome-wide distribution of rare variants in the same functional category to derive a gene-based Fisher’s exact *p*-value (two-tailed). Only variants with a MAF <0.5% in 1000G (2011 release with 1092 individuals) were included in the test. *P* values were corrected for multiple testing by applying a Bonferroni correction and adjusting for gene length. The top three genes with rare LoF variants are shown in Table 2. *ITGAE* (NM_002208.4) and *CUBN* (NM_001081) were suggestive candidates for further screening. *ITGAE*, also known as *CD103*, encodes an alpha integrin involved in tissue specific retention of T lymphocytes at the basolateral surface of intestinal epithelial cells and is a possible accessory function for the activation of epithelial cells [32,33]. Both novel nonsense SNVs in *ITGAE* (c.2962G>T, p.Glu988X and c.314T>A, p.Leu105X) were validated in the two sequenced exomes from FAM014 and Neu7058, and were subsequently tested for segregation in the two families. The c.314T>A (p.Leu105X) variant was present in four individuals in Neu7058, three of whom were non-disease cases. In FAM014, the c.2962G>T (p.Glu988X) variant was not observed in any other affected individuals, but only in one unaffected individual. Neither mutation segregated with disease. *CUBN* (cubilin) is located on chromosome 10p21.1 and is expressed within the epithelium of the intestine where it acts as a receptor for intrinsic factor-vitamin B (12) complexes [34]. Three novel nonsense SNVs in *CUBN* were observed in three separate individuals: c.4459C>T (p.Arg1487X), c.5428C>T (p.Arg1810X) and c.6359G>A (p.Trp2120X). Direct Sanger sequencing confirmed all variants, however the size of the coding region was too large (11,494bp, 67 exons) to be included in the candidate gene targeted resequencing assay.

**Table 2:** Top three most significant genes for the aggregate test for rare LoF variants only between cases (one per multiply affected family) and controls (with MAF <0.5% in 1000G).

**Gene**	**Number of rare alleles in controls (n = 220)**	**Number of rare alleles in cases (n = 41)**	**Fisher *p* value**
*ITGAE*	0	2	0.027
*TEX14*	0	2	0.027
*CUBN*	2	3	0.043

In a second analysis, we restricted our burden test to LoF variants in immune genes (Table 3) owing to the strong candidate status of these variants for an implication in immune related diseases. We selected seven genes from Table 3 for resequencing, based on known immune role and cDNA size: *CD1C, CERK, CRLF3, IKZF3, CD180, EB13* and *IFNW1*. Immune genes were defined as being present in the Gene Ontology list for immunologically important genes (see S5 Table for candidate gene immune functions and any known association or relevance to other autoimmune or gut diseases).

**Table 3:** Top 15 most significant genes for the aggregate test for rare LoF variants in immune genes between cases (one per multiply affected family) and controls (with MAF <0.5% in 1000G).

**Gene**	**Number of rare alleles in controls (n = 220)**	**Number of rare alleles in cases (n = 41)**	**Fisher *p* value**
*CD1C* *	0	3	0.005
*CERK* *	0	3	0.005
*CRLF3* *	0	3	0.005
*DDR1*	2	4	0.010
*HLA-DOA*	4	5	0.012
*ZFYVE16*	4	5	0.012
*IKZF3* *	1	3	0.016
*RPS6KA2*	1	3	0.016
*CDH17*	3	4	0.020
*LPP*	5	5	0.020
*CD180* *	0	2	0.022
*CTGF*	0	2	0.022
*DNM1L*	0	2	0.022
*EB13* *	0	2	0.022
*IFNW1* *	0	2	0.022

* Candidate genes selected for deep amplicon resequencing.

### 2. Non-parametric SNV-based linkage analysis

A model-free linkage approach was applied to define shared chromosomal intervals containing potential high-risk variants in affected individuals [35]. We hypothesised that causal variants would be present in candidate linkage regions and shared by sequenced members of each family. Sixty-six cases and 119 related controls from twelve coeliac pedigrees were genotyped on the ImmunoChip array (Fig. 1, pedigrees are illustrated in S3 Fig.). After applying quality control and pruning to keep common variants not in linkage disequilibrium, 3,700 polymorphic SNPs remained. We used Merlin to perform multipoint non-parametric analysis [35]. A non-parametric linkage test allowed no assumptions to be made about the disease model, with the hypothesis that high-risk alleles will be shared identical by descent (IBD) under the null of no linkage. Subjects were classified as affected, unaffected or unknown affection status according to pedigree records obtained by P. Ciclitira, S. Neuhausen and Coeliac UK members. The minimum *p*-value assuming perfect segregation of the risk variant in affected individuals was estimated using the non-parametric linkage (NPL) function of Merlin. The NPL statistic reported here, or *p*- value, reflects IBD alleles shared evaluating non-random segregation at chromosomal locations. The power of the linkage approach varies greatly depending on the family being considered (Table 4 and Methods). The smaller families (BD, H and BRE) are too small to reach the *p* < 0.01 threshold individually (but they contribute to the combined NPL statistic across all families). The larger families (SDY, FAM014 and FAM063) provide close to or over 75% power individually at our selected *p* <0.01 threshold. The power when analysing all twelve families jointly is 50%.

**Table 4:** Summary of non-parametric linkage results in twelve multiply affected disease pedigrees.

**Family ID**	**Minimum observed linkage p genome-wide**	**Power (p < 0.01, see Methods)**	**Size (Mb) of linkage region at p < 0.01**	**Number of rare, LoF and non-synonymous variants in linkage region**
DA	0.005	0.22	25.47	6
BRK	0.0011	0.24	40.08	3
BRE	0.0004	0.02	23.76	1
HMN	0.005	0.41	71.63	3
BD	0.05	0	0	0
BR	0.004	0.17	46.04	0
BUT	0.003	0.43	34.34	0
SDY	0.03	0.75	0	0
FAM008	0.0002	0.22	29.01	0
FAM063	0.0008	0.84	52.42	2
FAM014	0.005	0.75	26.98	3
H	0.05	0	0	0

Summary of linkage data for the twelve families included in the NPL analysis. The linkage *p*-values were computed using Merlin [33]. The power was assessed using simulations (see Methods).

Of the six families sufficiently large enough to produce NPL *p*-values < 10^−4^ (based on Merlin estimates of minimum possible NPL *p*-values), three reached these minimum observed linkage *p*-values for at least one locus: BRE, FAM008 and FAM063. This number was compatible with simulations under the null, which indicates an absence of enrichment of significant linkage *p*-values in these families. Although this may not be genome-wide significant it was our intention to identify loci with some evidence of sharing in which we would expect to find rare disease causing variants and thus pursued these linkage intervals further. An NPL analysis combining all families only highlighted the 6p21.32 locus, which contains the well-established HLA locus risk factor. We attempted to prioritise families with a low HLA risk as potentially harbouring rare non-HLA high-risk disease mutations, but no low risk HLA genotypes were observed in coeliac cases: 46% of affected individuals in our cohort were HLA-DQ2.5 homozygote or heterozygote, and 100% had at least one copy of DQ2.5 or DQ8. Overall 74.5% (70/94) of unaffected individuals carried either HLA-DQ2.5 or HLA-DQ8 homozygous or heterozygous genotypes, which is larger than the overall population (47.5% of general population carry DQ2.5/DQ8 molecules), as may be expected in unaffected relatives of affected individuals. Furthermore, of the 74.5% DQ2.5/DQ8 genotypes from unaffected individuals, 18.6% were from individuals who married into the family (13/70).

Linkage regions with *p* < 0.01 were assessed for exonic variants and filtered using the following settings: i) MAF <0.5%, ii) only variants predicted to be damaging, iii) regions without duplications. In total, 18 rare nonsynonymous coding variants were identified in linkage regions (Table 5). No rare indels were observed under linkage peaks. To test whether the identified variants were present on the same haplotype in all affected members of the linkage pedigree, all variants were Sanger sequenced in each affected subject. Ten of 18 nonsynonymous SNVs were validated in all affected individuals from five pedigrees, indicating presence on the same ancestral haplotype. Eight candidate genes harbouring these variants were subsequently selected for candidate gene resequencing on the basis that variants in this genes were present on the same ancestral haplotype in linkage regions and not solely on if they were immune genes (S5 Table): *NLRC4, EPAS1, ARHGAP25, GRM4, TULP1, KCNJ16, MATL1*, and *ACOT8*.

**Table 5:** Nonsynonymous missense SNVs located in linkage regions (*p* < 0.01).

**Gene**	**Chr: position**	**SNV**	**PolyPhen Prediction**	**dbSNP132 ID/function**	**Cases validated/Cases tested**
*FAM179A*	2:29259543	c.2555T>C	-	rs72788155/missense	2/6
*NLRC4* *	2:32474767	c.2166T>G	Probably damaging	-	6/6
*EPAS1* *	2:46607609	c.1798G>A	Possibly damaging	-	7/7
*STON1*	2:48809609	c.1837C>G	Probably damaging	-	2/7
*ARHGAP25* *	2:69040504	c.739G>A	Probably damaging	rs61758703/missense	4/4
*IQGAP2*	5:75969341	c.3136G>T	-	-	1/6
*DMGDH*	5:78293933	c.2573A>C	Probably damaging	-	4/6
*KIF13A*	6:17826085	c.1700A>C	-	-	5/5
*BRD2*	6:32942277	c.68G>A	Probably damaging	rs55650502/missense	4/6
*GRM4* *	6:34101193	c.81G>A	Benign	-	5/5
*TULP1* *	6:35471412	c.1247G>A	Probably damaging	-	5/5
*SYTL2*	11:85445365	c.1004C>G	Probably damaging	rs74718633/missense	2/6
*ABCA9*	17:67039672	c.758C>T	Possibly damaging	-	4/4
*KCNJ16* *	17:68129412	c.1184A>G	Benign	-	4/4
*SDK2*	17:71431712	c.1072C>T	-	-	1/4
*MALT1* *	18:56402558	c.1567G>A	Probably damaging	-	6/6
*ACOT8* *	20:44470575	c.862C>T	Probably damaging	-	4/4
*EYA2*	20:45808514	c.1267C>T	Possibly damaging	-	1/4

* Candidate genes selected for deep amplicon resequencing

### 3. Large-scale candidate gene resequencing

Candidate gene selection for follow-up deep amplicon resequencing was based on the following criteria: i) shared exonic variants in related exomes including positive segregation in familial disease cases, ii) a higher burden of exonic variants in any one gene in cases or controls, iii) genes in linkage regions, iv) genes with an interesting or disease-related immune function. S4 and S5 Tables list candidate genes selected from the various analytical strategies, based on the set criteria.

We initially sequenced 2,304 coeliac cases and 2,304 controls in a 506-amplicon PCR assay using Fluidigm Access Array technology. Three libraries contained excellent barcode coverage across 1,536 10bp sequences, with 99.6% of the 1,536 barcodes producing pass-filter read numbers. These were between 0.013% and 0.13% of total pass filter reads per lane. Most failing barcodes (48 out of 68) were water (negative control) samples. Amplicon evenness was excellent with many genotypes requiring down-sampling of 250 bases per site per sample (S4 Fig.). A filter of >20 mean depth per sample was applied to call a variant. 3.47% of 55,807 unique bases had <20 mean depth per sample and were all accounted for by 18 amplicons that failed PCR. Thirteen out of the 18 failed amplicons had high GC content (between 63% and 89%).

The high coverage data enabled stringent filtering on call rate per sample, per variant site and allelic balance. After stringent quality control, the final dataset comprised 4,478 phenotyped individuals (2,248 disease cases and 2,230 controls) and 1,335 unique SNVs and indels with a genotype call rate of 99.98%. Of the genotype calls, 99.98% had a read depth >40 and 97.4% had a read depth >100. Of the 1,335 variants, 1,200 variants were rare (MAF of <0.5% based on 2,230 controls), 502 variants were observed in published datasets (dbSNP137 containing all 1000G pilot data plus phase 1 low coverage sites and National Heart, Lung and Blood Institute exome data from 6,503 samples), and 833 variant sites were novel (not observed in controls or databases). The number of coding variants (defined as one that is present in the coding region) per gene was assessed (Table 6). Of the 1,335 variants, 939 were in protein-coding regions of 24 genes and of these 91.7% were rare (MAF in 2,230 controls, <0.5%). 60% of all coding variants were novel when compared with published datasets. No common or low frequency variants were seen at novel sites (mean MAF 0.0014%). Overall, 60 rare LoF variants (nonsense, codon indel, frameshift, and splice site; based on GENCODE v14 annotations) were identified across 20 genes; four genes harboured no such variants.

**Table 6:** Number of coding, rare and LoF variants across 24 candidate genes selected from exome sequencing of 75 individuals from multiply affected families.

**Gene**	**Number of variants in coding regions**	Number of rare (MAF* <0.5%) in coding regions	Number of rare (MAF* <0.5%) and LoF
*ACOT8*	29	27	5
*ARHGAP25*	40	34	2
*C1QBP*	9	8	2
*CD180*	48	43	4
*CD1C*	28	26	3
*CERK*	54	48	4
*CRLF3*	23	20	2
*EBI3*	28	25	2
*EPAS1*	59	55	3
*GRM4*	69	65	0
*HAS1*	61	56	1
*IFNW1*	17	16	0
*IKZF3*	29	27	1
*IL12RB1*	60	52	4
*KCNJ16*	36	35	3
*MALT1*	21	20	3
*MAP4K2*	34	33	2
*NLRC4*	64	61	3
*RAF1*	27	26	2
*TNFRSF10A*	46	42	5
*TNFRSF13B*	42	34	4
*TNFRSF21*	39	38	0
*TRAF4*	30	28	0
*TULP1*	46	42	5

* MAF as defined in controls.

Data quality was confirmed by a number of steps. One control sample was genotyped 42 times (on different 48-sample microfluidic chips); the genotype call error-rate was two non-consensus genotype calls of 1,335 called genotypes (0.0018%). A quality control step measuring TiTv ratios for expected human mutation types was 2.99 (3.18 for singletons) for coding-region variants, 2.86 (3.13 for singletons) for rare variants, and 2.69 (2.90 for singletons) for novel variants. For novel coding-region variants the TiTv ratio was 2.78 (2.89 for singletons). Sanger sequencing validation analysis was performed on all nonsense (17) and frameshift (11) variants. One variant consistently failed PCR and two variants (a frameshift indel and a nonsense SNV) were false positive (false positive rate = 7.4%).

#### 3.1 Single SNV and gene-burden tests in resequenced coding regions

For single variants, and similarly for gene burden tests, given an allele frequency in controls equal to 0.5%/1%/5%, the sample size of the follow-up resequencing study (2,248 cases and 2,230 controls) provides 90% power (at *p* < 10^−4^) for odds ratio 3.42/2.48/1.57 (S5B Fig.). A first attempt to identify any low frequency or rare variants of larger effect was performed for each coding-region variant in a Fisher’s exact single-variant association analysis. 135 variants common in controls were removed from the test (MAF <0.5%). A significant *p* value of 6 × 10^−5^ was chosen to account for multiple testing on 939 rare coding variants. No single SNV associations were observed (the most significant *p* value was 0.012). A gene-based C-alpha test [36], a sequence-kernel association test [37] and tests to identify excess rare variants in cases, collectively (Burden test) and uniquely (Uniq test), were subsequently performed on all coding variants across 24 genes. Rare functional variants included in the tests were defined as MAF <0.5% in 2,230 controls and predicted as nonsense, frameshift, codon indel and splice site. Here, a Bonferroni *p* value of <1 × 10^−3^ was selected based on the number of transcripts tested, and not the number of genes, as some genes had multiple transcripts. No significant *p* values were observed in any test for novel or known variants (Table 7 shows genes with top five *p*-values across all gene-based tests).

**Table 7:** Top five *p*-values for multiple rare variant gene-based tests across all protein-coding variants (novel and known) in 24 candidate genes (case control analysis in 2,248 cases and 2,230 controls).

**Gene**	**Transcript**	**Rare variant test**	**Number of variants in test**	**Test statistic *p* value**
*CERK*	NM_022766	C-Alpha	48	0.022
*ARHGAP25*	NM_001007231	C-Alpha	34	0.118
*HAS1*	NM_001523	C-Alpha	56	0.119
*IL12RB1*	NM_005535	C-Alpha	52	0.229
*TNFRSF13B*	NM_012452	C-Alpha	34	0.275
*CERK*	NM_022766	SKAT	48	0.002
*ARHGAP25*	NM_001007231	SKAT	34	0.096
*HAS1*	NM_001523	SKAT	56	0.126
*IL12RB1*	NM_005535	SKAT	52	0.188
*CD1C*	NM_001765	SKAT	27	0.263
*EPAS1*	NM_001430	UNIQ	55	0.004
*CD1C*	NM_001765	UNIQ	27	0.044
*HAS1*	NM_001523	UNIQ	56	0.092
*IFNW1*	NM_002177	UNIQ	16	0.140
*RAF1*	NM_002880	UNIQ	26	0.229
*EPAS1*	NM_001430	Burden	55	0.007
*ARHGAP25*	NM_001007231	Burden	34	0.167
*TNFRSF21*	NM_014452	Burden	38	0.234
*CD1C*	NM_001765	Burden	27	0.240
*TNFRSF10A*	NM_003844	Burden	42	0.262

## Discussion

In this paper, we describe an investigation into whether rare mutations with high penetrance for CeD are identifiable in a familial dataset, possibly accounting for some of the missing disease heritability. We report an exome sequencing study in large multiply affected CeD families to locate novel (possibly rare) disease associated variants in 75 coeliac cases of Caucasian origin, where one, two or three related subjects per family were sequenced (from 55 families in total). We combined non-parametric linkage analysis (since the inheritance model of CeD in the pedigrees was uncertain) of our twelve largest UK pedigrees with the exome sequencing data. We then performed Fluidigm amplicon PCR and deep resequencing of 24 candidate genes in a large case-control cohort to allow complete dissection of rare variation in candidate genes in a large sample set.

Thousands of protein-coding mutations per individual were identified across each exome, posing a ‘needle in a haystack’ situation. Consequently, we sequenced multiple exomes within families to filter potential high-risk variants shared in a disease pedigree. Most candidate SNVs failed to segregate directly in CeD subjects and only one positive segregation test was observed in the BRK family, in which all CeD cases carried the nonsense c.184C>T (p.G62S) variant in *TNFRSF21*, although this was not deemed to be statistically significant. In eight genes, we observed shared nonsynonymous missense SNVs in related CeD subjects, with no segregation data available.

We then combined linkage analysis with the exome sequencing data to pinpoint causal functional variants in regions where excess allele sharing was evident (NPL methods identify alleles shared IBD). We were successful in identifying shared variants (present in all affected individuals) in linkage regions from five coeliac pedigrees. There was no overall excess of significant linkage *p*-values: the maximum linkage score was observed in three of the well-powered pedigrees, a result consistent with the expected null distribution. If such rare variants indeed exist, our inability to detect clear linkage signals might be explained by rare alleles shared identically by state (IBS) rather than by descent. This issue is, for example, observed in the SDY family where a proportion of married in individuals are carriers of the HLA risk alleles, explaining the lack of linkage signal at this well established locus (HLA genotypes illustrated in S3 Fig.). Replication of HLA linkage has also been unsuccessful in other CeD linkage studies due to lack of distinction between alleles IBD and IBS, low marker density and no differences in inheritance patterns between affected and unaffected members [38]. Another explanation could be the presence of sporadic cases not sharing a rare damaging variant shared by the majority of other cases in the family. In support of the linkage *p*-values obtained here, another study reported maximum NPL LOD scores of 1.9 at 10q23.1 and 16q23.3, and 1.5 at 11p11 with the same families tested in this study [39]. We attempted to prioritize families without any HLA high-risk variants (HLA DQ2.5 and HLA DQ8), but did not observe any in our twelve linkage family dataset. An alternative strategy might have been to prioritize families with low disease risk by accounting for all known established loci, a strategy that has been suggested in the literature [26].

To analyze the sequencing results of the 24 candidate genes in 2,248 cases and 2,230 controls, gene-based tests, in which multiple rare variants in the gene region are jointly analyzed to aggregate all signals, were performed to better detect the combined effects of multiple variants, given the evidence that multiple rare variants can have a collective effect on disease risk [17, 40]. The gene with the most significant *p*-value (*p* = 0.004 in a uniq case-control allele test and *p* = 0.007 in a burden test) was *EPAS1*, a transcription factor that produces the hypoxia-inducible factor 2-alpha protein and which has been implicated in hyperglycemic mice [41]. This gene may warrant further follow-up. Further experimentation with *CUBN* also remains to be carried out to investigate possible rare variants in CeD subjects; this gene was too large to incorporate into the Fluidigm resequencing assay reported in this paper.

While our exome sequencing strategy identified suggestive variants for disease association, we found no significant association between rare or low frequency LoF coding variants (identified in our large multiply affected families) with disease in our candidate gene resequencing study. A limitation of this approach is the imperfect power to prioritize such variants in the first stage of our study. To increase our chances to detect possible causal variants, we combined several strategies in the discovery stage (linkage, burden test and shared rare variants between distantly related cases). Owing to the large number of private mutations observed in the exome data (mean ∼ 650 per exome), a strict selection criterion was necessary and true causal variants in novel genes may have been missed as a consequence of this. Imperfect knowledge of genes implicated in immune pathways is another limitation of our selection process.

Notably, no rare variants were identified at the *SOCS1-PRM1-PRM2* locus (a rare variant, imm_16_11281298, was previously identified in the CeD ImmunoChip study at this locus; *p* = 1.3 × 10^−4^, MAF = 0.004, OR of 1.70 [6]), or any other coeliac-associated GWAS regions, indicating: 1) rare variants clustering in families are different to population-wide common variants predisposing to disease risk (as observed for *TNFRSF21* where the novel variant c.184C>T (p.G62S) segregated in all affected individuals in family BRK, but no rare variants in this gene were associated with disease risk in our candidate gene resequencing study) or 2) lack of power to identify rare variants.

Although we tested for presence of rare variants in familial (and not sporadic) subjects, these findings do provide support for other studies reporting a lack of rare variant associations in complex diseases: a recent resequencing study of 25 GWAS risk genes from six autoimmune diseases in 42,000 subjects concluded that rare coding mutations play a negligible role in the autoimmune diseases under investigation [8], and a similar study in psoriasis, with a much larger exome sequencing discovery set (781 cases), found no evidence of rare nonsynonymous variants at the resequenced candidate genes in 9,946 cases and 9,906 controls [42]. Studies are now indicating that many common variants of small effect may contribute to disease susceptibility, for example in high-density lipoprotein cholesterol where 61.8% of contribution to cholesterol levels is from common variants, compared to 7.8% from rare variants [43]. With a limited sample size for the discovery sequencing method (n = 75 exomes), the dataset here does not provide any evidence that rare variants in familial CeD subjects account for a proportion of the disease heritability in CeD but there may be additional rare variants in the coding and non-coding regions (or perhaps, specific for sporadic disease) that were not investigated here that may explain this. Rare variation in *EPAS1* and *CUBN* (not sequenced here) might be further investigated for their potential roles in CeD.

## Materials and Methods

### Subject selection

Affected coeliac individuals were diagnosed according to standard clinical, serological and histopathological criteria, including small intestinal biopsy [44]. Written informed consent was obtained from all subjects. DNA samples were from blood or saliva, collected from Coeliac UK charity members and approved by the Oxfordshire B Research Ethics Committee (UK). UK coeliac pedigrees DA, BRK, BRE, HMN, BD, BR, BUT and SDY were provided by P. Ciclitira at St Thomas’ Hospital London and approved by the St Thomas’ Hospital Ethics Committee (UK). Other European coeliac families NEU4768, NEU4801, NEU7017, NEU7058 and NEU4735 were provided by S. Neuhausen at Beckman Research Institute at the City of Hope, California and approved under the Office of Human Research Subjects Protection, City of Hope (USA). One Swedish coeliac family, NAL108, was provided by Å, Naluai at Gothenburg University, Sweden and approved by the regional ethics board in Gothenburg.

Since this study was completed when exome sequencing was a relatively new method, control exomes were provided by three separate institutions at different stages of experimental work: R. Trembath at Kings College London provided 54 control exomes from ultra-rare diseases approved by the South London Research Ethics Committee (UK), D. Nickerson at University of Washington provided 47 control exomes from the Environmental Genome Project approved by the National Institute of Environmental Health Sciences Ethics Office (USA) and H. Hummerich at Institute of Neurology, University College London provided 220 control exomes from prion disease samples approved by the National Hospital for Neurology and Neurosurgery Local Research Ethics Committee (UK).

### Exome sequencing

Exome library preparation and capture was performed at The Blizard Institute, Barts and the London. Five micrograms of genomic DNA from 75 coeliac individuals was prepared for Illumina high throughput sequencing and captured with 2.1 million exon probes in solution following NimbleGen’s EZ Human Exome In-solution protocols (version 1 contained 27.6Mb of coding DNA equating to ∼180,000 exons and version 2 contained 44.1Mb of coding DNA). In brief, genomic DNA samples were initially fragmented using the Covaris (settings: duty cycle 10%, intensity 5, cycle/bust 200, time 140s). The following steps were then performed on each fragmented DNA sample: end-repair, addition of an adenine base to 3’ end and ligation of Illumina paired-end sequencing adapters. Ligated samples were cleaned with solid phase reverse immobilization (SPRI, AMPure XP A63880) beads. Post SPRI-purified samples were then hybridized to exon probes in solution for 64–72 hours on a thermocycler. Hybridized samples were washed and eluted with Streptavidin Dynabeads (Invitrogen 653–05). A post capture quantitative PCR was performed on twelve reactions per eluate and two reactions with SYBR green I dye to measure fluorescence. The reaction was terminated before amplification reached the plateau curve (typically between 13 and 15 cycles), and two pools of five PCR reactions (not containing SYBR green I) were combined and cleaned with QIAquick PCR purification kit (Qiagen 28106).

Single exome libraries were sequenced on the Illumina GAII*x* with 76bp paired-end reads at Barts and the London Genome Centre. Sequenced reads were aligned to an indexed human genome (hg18) using the short read mapper Novoalign (v2.00.07), with gapped quality-aware alignment and settings -c 14 -H -k -a -o Soft. The Needleman-Wunsch algorithm was used for paired-end data. SNP calling was performed using Samtools single sample calling (v0.1.8) and default filtering options. Variants were annotated with SeattleSeq and then Annovar (RefSeq NCBI build 37, 1000G 2011 release, and dbSNP132 identifiers), released in 2010.

### Control exomes

Control samples for exome sequencing were participants in studies of early-onset dementias and included diagnoses of variant Creutzfeldt-Jakob disease (n = 87, average age of onset (AAO) 30 (range 14–62), 58% male), sporadic Creutzfeldt-Jakob disease (n = 49, average AAO 55 (range 15–85), 40% male), Alzheimer’s disease (n = 41, average AAO 55 (range 38–74), 43% male), frontotemporal dementia (n = 42 average AAO 55 (range 39–68), 59% male) and neurodegenerative syndromes in keeping with Huntington’s disease but negative for an expansion in huntingtin (n = 5, average AAO 42 (range 23–60), 100% male). Patients were recruited by the NHS National Prion Clinic and the Dementia Research Centre or others at University College London Hospitals NHS Trust. The research study was approved by the National Hospital for Neurology & Neurosurgery Research Ethics Committee. Target enrichment was performed using the Agilent 50M exome sequencing kit and the bioinformatics analysis was done using the same settings (i.e. alignment/calling/annotation) as the CeD exomes.

### ImmunoChip genotyping and non-parametric linkage analysis

All available subjects from twelve coeliac pedigrees (selected from the 55 families in our dataset) were genotyped on the Infinium HD ImmunoChip custom array designed by Illumina (containing 196,543 polymorphisms (718 small indels and 195,806 SNPs)), according to Illumina’s protocols. Genotyping was performed at Barts and the London Genome Centre. In brief, genomic DNA was whole-genome amplified without PCR by overnight incubation. After fragmentation, precipitation and resuspension, each DNA sample was hybridized to the custom beadchip in a capillary flow-through chamber. Non-specific DNA was washed away and then stained for single base extension of the oligonucleotides present on the chip. All beadchips were scanned on the Illumina iScan at the Institute of Child Health, University College London. NCBI build 36 (hg18) mapping was used.

To select for polymorphic SNPs for subsequent linkage analysis, a Hardy-Weinberg filter of 0.001, a MAF of 0.2 and a differential missingness filter of 0.001 was applied in PLINK [45]. SNPs were then pruned using an r^2^ threshold of 0.2, leaving 3,700 SNP markers for linkage analysis. Multipoint NPL analysis was performed with Merlin linkage analysis software [35] based on the Kong and Cox score statistics comparing alleles shared IBD for all affected individuals [46]. An exponential model was selected to prove the hypothesis that rare variants of large effect size result in large increases in allele sharing in families compared to common variants of small effect size.

### Assessing the contribution of known CeD risk alleles

To assess the contribution of known CeD risk alleles on the expected number of individuals within a subset of the families (the twelve largest families with ImmunoChip data available) we examined 57 such variants (including a SNP tagging HLA DQ2.5) and used previously described estimates of their allele frequency in controls and their effect size on CeD risk (S2 Table) assuming additivity within and between loci. Analyses were conducted using the R-package Mangrove [26] with a population prevalence of CeD of 1%. The genotypes of ungenotyped individuals within the pedigrees were sampled (n = 1,000 per family) conditional on the genotypes of genotyped relatives and standard risk prediction, implemented in Mangrove, was used to determine the distribution of the expected number of affected individuals within each family. This number was summated across all families for each of the 1,000 simulations and the resulting distribution compared with the observed number of affected individuals in all families.

### Power study for linkage analysis

To assess the power of NPL analyses either in each of the twelve families, or when combined, we used a Monte Carlo simulation procedure. Genotype data were first simulated for chromosome one for each family under the null (assuming no linkage to disease) using Merlin [35]. To enable the number of affected individuals that had inherited each allele identically-by-descent to be ascertained—a value not directly provided by Merlin—each allele was tagged with a marker at the same genetic position, which uniquely identified the founder pedigree member from which it derived. These tags were removed prior to linkage *p*-values being calculated using Merlin with a non-parametric exponential model as employed for locus identification. A single point on the chromosome was then selected and the minimum *p*-value within a 5 cM window of that point and the corresponding maximum number of affected individuals that had inherited a founder allele identically-by-descent within the window were identified. This process was repeated 1,000 times per family. The power was then calculated by computing the proportion of *p*-values across the 1,000 simulations achieving the <0.01 threshold, with each simulation weighted by its probability under the alternative. That alternative hypothesis scenario was set up such that disease probability is 80% for carriers and 3% for non-carriers (a number slightly greater than the baseline population prevalence of the disease to account for a possible polygenic effect).

### Fluidigm Access Array assay design and wet-laboratory method

Fluidigm designed PCR primers for all RefSeq exons of 24 candidate genes totaling 506 amplicons containing exonic sequences. Amplicons were selected to be 150–200bp in size. The design covered all exons, excluding any 5’ or 3’ un-translated regions. 21 out of the 24 target genes had 100% total coverage of all exon amplicons. There was minor primer design dropout at *MALT1, MAP4K2* and *IL12RB1*, however amplicons for these genes still covered 98.8%, 99.3% and 97.99% of exons, respectively. The total length of overlapping amplicons was 96,581bp, 68,494bp with primers removed and overlapping, and 55,807bp unique (non overlapping and primers removed). One Fluidigm Access Array was intended to multiplex PCR 48 samples with 506 primer pairs (11-plex assay per well). The 4,608 sample set included water negative controls.

50ng genomic DNA was PCR amplified in a multiplexed Fluidigm Access Array microfluidics system, at Barts and the London Genome Centre, following Fluidigm’s protocols. Individual per sample per primer pool PCR reactions took place in 35nl reaction chambers. PCR amplicons from a sample were pooled and barcoded (via PCR) with one of 1,536 unique 10bp sequences (Fluidigm unidirectional sequencing protocol). An equal number of cases and controls were combined to create one 1,536 multiplex library. Three libraries were generated in total. Libraries were initially sequenced on an Illumina MiSeq (50bp, single-end) for quality control of individual barcodes and to optimise loading concentrations and cluster density targets for Illumina HiSeq sequencing. Libraries were then sequenced (one library per sequencing lane) with 101bp paired-end reads and a 10bp index on the Illumina HiSeq 2000 at the Biomedical Research Centre at Guy’s Hospital. For three HiSeq 101bp, 10bp index, paired-end sequenced libraries, >93% reads passed filter with on target cluster densities between 640–775 k/mm2.

### Bioinformatics for candidate gene resequencing

Individual sequenced samples were demultiplexed by Ilumina CASAVA software, allowing zero mismatches per 10bp barcode. In brief, data analysis processes from 9,216 fastq files included: 1) PCR amplicon trimming of 5’ end of individual reads using a modified version of Btrim [47], 2) read mapping of trimmed sequences to hg19/build37 of an indexed reference genome using Novoalign v2.07.18 with gapped quality-aware alignment, 3) local realignment around known (1000G) and sample level novel indels, 4) base quality score recalibration, 5) SNP and indel calling, and 6) variant annotation. Steps 3 to 6 were performed using GATK 2.4–7. Settings used for SNP and indel calling and variant annotation were as previously described [8]. The variants used were restricted to sites that passed standard GATK filters to eliminate SNPs with strand bias, low quality of read depth, homopolymer runs and SNPs near indels. Variants with an average depth >20 and a quality score >80 were required. SNP genotypes were called at all 68,494 bases of amplicon sequence. Non-reference genotype sites were identified across all samples and VCF files containing polymorphic variant sites and samples were combined for use with PLINK/SEQ v0.09. Annotation was performed with GENCODE V14 gene definitions. Coding variants were identified as present in coding regions, and rare functional variants were identified based on nonsense, splice, esplice (splice site in the first or last two intronic bases), frameshift indel, codon indel (3n indel), readthrough, and start lost predictions. PLINK/SEQ v0.09 was used to perform all single variant and gene-based association analyses and for determination of TiTv statistics. Quality control steps included the removal of: water samples (negative controls), samples with low call rates across all SNVs, SNVs with low call rates across all samples. Samples discordant with ImmunoChip genotypes and/or with known gender or genotype mismatch issues from previous GWAS were excluded [6]. Samples with known duplicates or relatedness (as distant first cousins) were excluded. The initial PLINK/SEQ project contained 2,292 polymorphic variants and 4,608 samples. A SNP and indel call rate of 97.7% and individual genotyping call rate of 97% (across all SNPs and indels) was applied. All heterozygous calls were required to have an allele balance between 25% and 75%. The mean allele balance at all heterozygous sites was 0.49±0.12. The mean ± two standard deviations was 24% and 73%, similar to the 25%-75% allele balance used here and in Hunt et al [8]. The gene-based C-alpha test used for rare variant association analysis allowed for both risk and protective effects for rare functional variants [36] and the SKAT test is a variance-component test that aggregates individual score statistics by assigning weights for each SNP to perform [37].

### Capillary Sanger sequencing

We validated variants by direct resequencing using a standard Sanger method. Candidate variants were Sanger sequenced in exome-sequenced individuals, and a control sample, for validation. If the variant was a true positive and present in all the exomes it was found in (i.e. in both first cousins from a pedigree), it was tested for segregation in the entire family, if DNA was available. The same was applied to candidate variants from NPL results. From the Fluidigm candidate gene resequenced variants, all samples with rare variant allele genotypes, and a control sample, were sequenced for 27 sites selected.

## Supporting Information

S1 Fig Mangrove analysis to quantify the expected number of affected individuals based on CeD prevalence and risk allele carriage. Histogram showing the distribution in the number of affected individuals expected across the twelve families used in linkage analyses based on the effect of 57 CeD risk variants (S2 Table) from 1,000 simulations using the R-package Mangrove [26]. The blue dotted lines show the 95% confidence intervals on the expected number of affected individuals (6–36) as compared with the number observed (76, red vertical line).
(TIFF)

S2 Fig Number of non-reference SNV calls per exome (n = 75) and corresponding average read depth. (TIFF)

S3 Fig Pedigree structures with HLA genotypes for twelve CeD pedigrees. Family BD. Family BR. Family BRE. Family BRK. Family BUT. Family DA. Family 008. Family 014. Family 063. Family H. Family HMN. Family SDY. All subjects were genotyped on the Illumina ImmunoChip array. Sample names in black were exome sequenced. Sample names in blue were included in the linkage test. HLA genotypes are shown below the sample name. X denotes ‘other genotype’.
(PDF)

S4 Fig Mean depth of coverage per sample for 100 random targeted re-sequenced samples. Depth data was produced with a random 100 of 4,478 post quality control samples. GATK settings applied were: minimum base call quality 16, mapping quality >40 and down-sample reads to 250x per sample.
(TIFF)

S5 Fig Power calculations for the exome discovery and candidate gene deep amplicon resequencing studies. A. Power calculation for single variant association testing in the discovery exome sequencing dataset for 41 exome cases (one per multiply affected family) and 220 controls. B. Power calculation for single variant association testing in 2,248 cases and 2,230 controls included in the deep amplicon resequencing study. Three scenarios are shown reflecting different allele frequencies in controls: 2% (blue line), 1% (red line) and 0.5% (black line). Y-axis shows power to detect association at p<10^−4^ as a function of the odds ratio parameter.
(PDF)

S1 Table Total numbers of affected cases per family and numbers of sequenced coeliac cases per family. The numbers of affected, unaffected and status unknown individuals out of the total number of individuals is shown per family, separated by slashes. A ‘-‘ denotes unknown number of individuals. *Pedigrees selected for non-parametric linkage analysis.
(DOCX)

S2 Table 57 CeD associated variants examined in the Mangrove analysis. The highest ImmunoChip-associated variant for CeD was selected per locus. The allele frequency of the risk allele (AF) in controls and the odds ratio (OR) for CeD, as reported by [6] are shown. Position refers to NCBI build 37. ^^^ Variant as reported in the CeD ImmunoChip study [6].
(DOCX)

S3 Table Summary statistics for 75 CeD familial exomes. One sample per line. *Samples sequenced twice due to initial poor capture and/or sequencing run.
(DOCX)

S4 Table Candidate genes for deep amplicon resequencing selected from exome data of 75 CeD individuals from multiply affected families. *Information taken from Ensemble genome Browser, release 71.
(DOCX)

S5 Table Immune function and autoimmune disease associations of 24 candidate genes selected for deep amplicon resequencing. Immunologically important genes downloaded from Gene Ontology (http://wiki.geneontology.org/index.php/Immunology) and used as a guide to select genes in immune mediated pathways for the candidate gene resequencing study. Immune functional information collected from NCBI (http://www.ncbi.nlm.nih.gov/gene/), T1Dbase (http://t1dbase.org) and Online Mendelian Inheritance in Man (OMIM; http://www.ncbi.nlm.nih.gov/omim) websites. Association with autoimmune diseases at the genome-wide level gathered from the Catalog of Published Genome Wide Association Studies (http://www.genome.gov/).
(DOCX)
