# Supplementary figures and images for: Exome Sequencing of 75 Individuals from Multiply Affected Coeliac Families and Large Scale Resequencing Follow Up
Source: PLoS One. 2015 Jan 30;10(1):e0116845. doi: 10.1371/journal.pone.0116845 (PMC4312029; doi:10.1371/journal.pone.0116845)

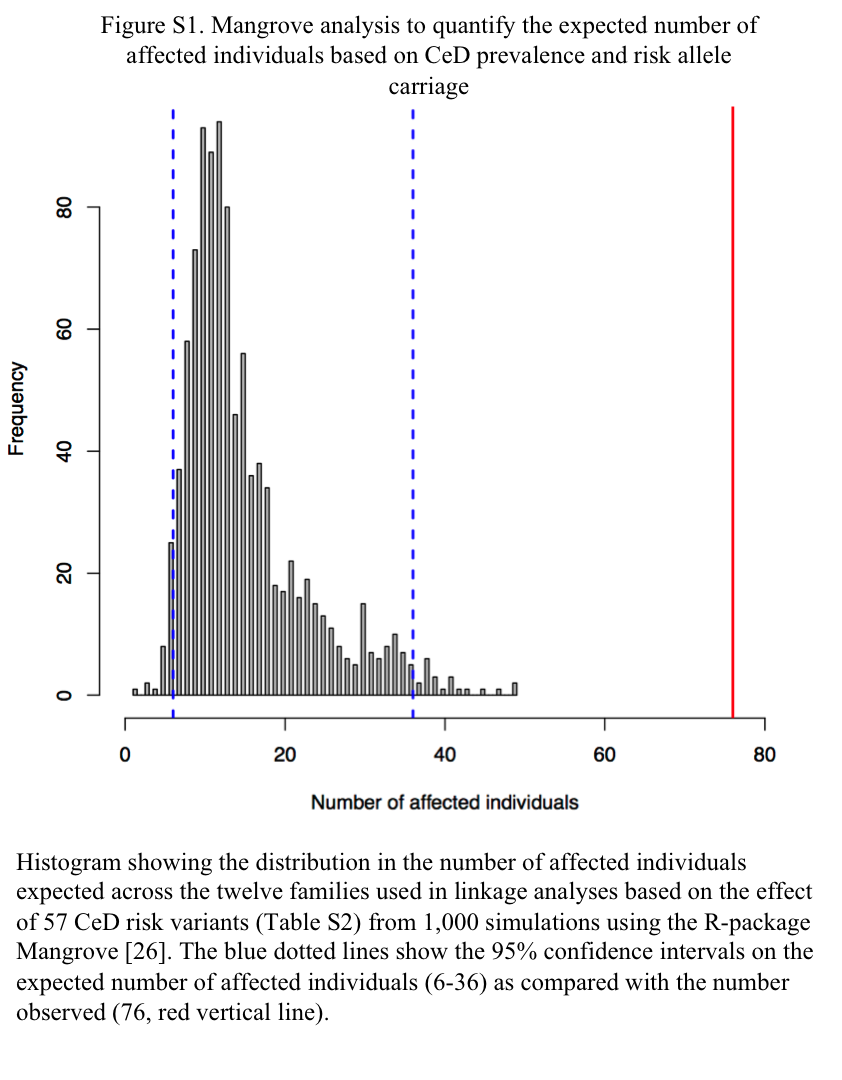

Supplement: S1 Fig — Histogram showing the distribution in the number of affected individuals expected across the twelve families used in linkage analyses based on the effect of 57 CeD risk variants (S2 Table) from 1,000 simulations using the R-package Mangrove [26]. The blue dotted lines show the 95% confidence intervals on the expected number of affected individuals (6–36) as compared with the number observed (76, red vertical line). (TIFF) [file pone.0116845.s001.tiff]

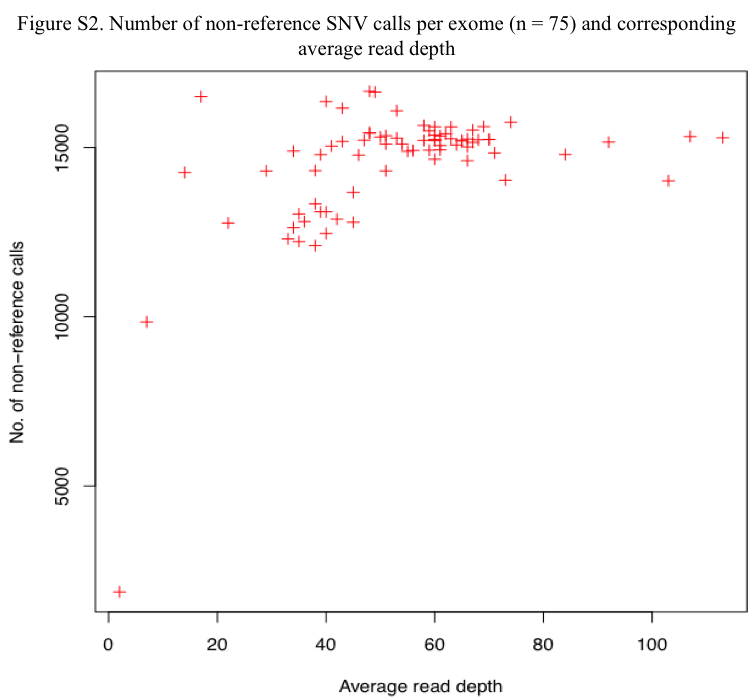

Supplement: S2 Fig — (TIFF) [file pone.0116845.s002.tiff]

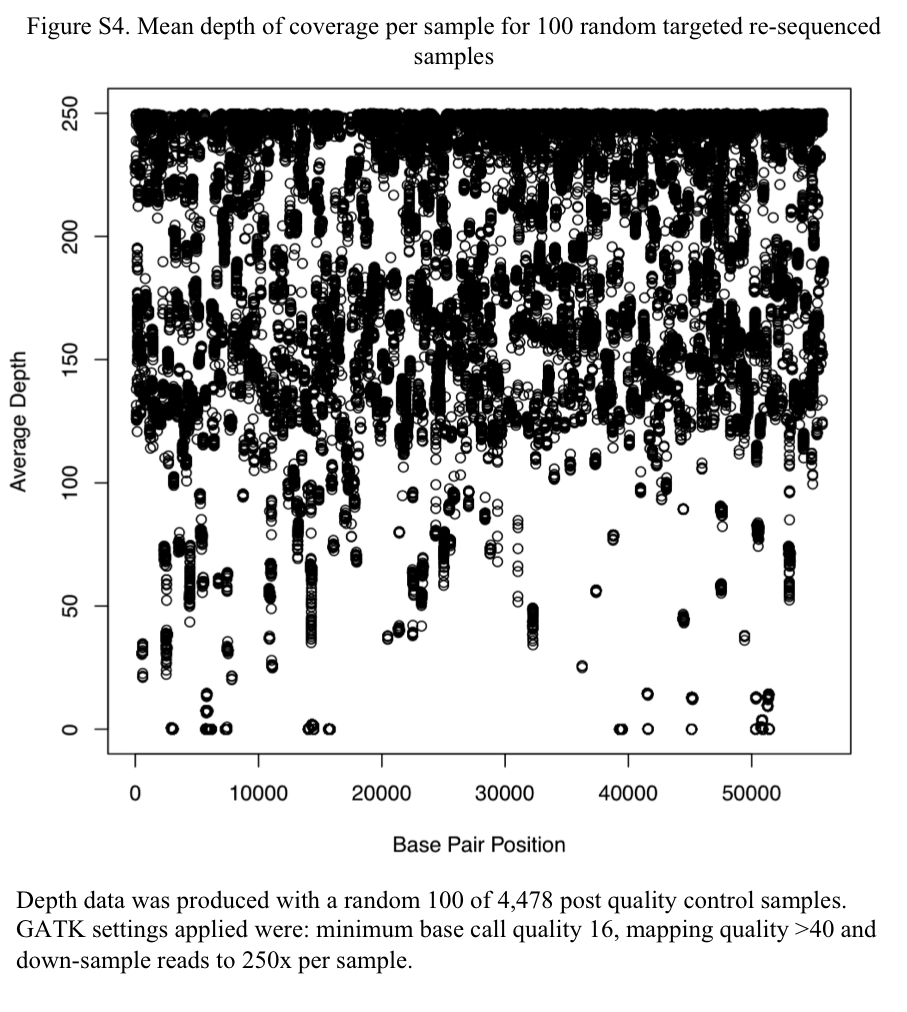

Supplement: S4 Fig — Depth data was produced with a random 100 of 4,478 post quality control samples. GATK settings applied were: minimum base call quality 16, mapping quality >40 and down-sample reads to 250x per sample. (TIFF) [file pone.0116845.s004.tiff]
