# Supplementary material for: Exome Sequencing of 75 Individuals from Multiply Affected Coeliac Families and Large Scale Resequencing Follow Up
Source: PLoS One. 2015 Jan 30;10(1):e0116845. doi: 10.1371/journal.pone.0116845 (PMC4312029; doi:10.1371/journal.pone.0116845)
Supplement: S3 Fig — Family BD. Family BR. Family BRE. Family BRK. Family BUT. Family DA. Family 008. Family 014. Family 063. Family H. Family HMN. Family SDY. All subjects were genotyped on the Illumina ImmunoChip array. Sample names in black were exome sequenced. Sample names in blue were included in the linkage test. HLA genotypes are shown below the sample name. X denotes ‘other genotype’. (PDF) [file pone.0116845.s003.pdf]

Figure S3. Pedigree structures with HLA genotypes for twelve CeD pedigrees

Family BD

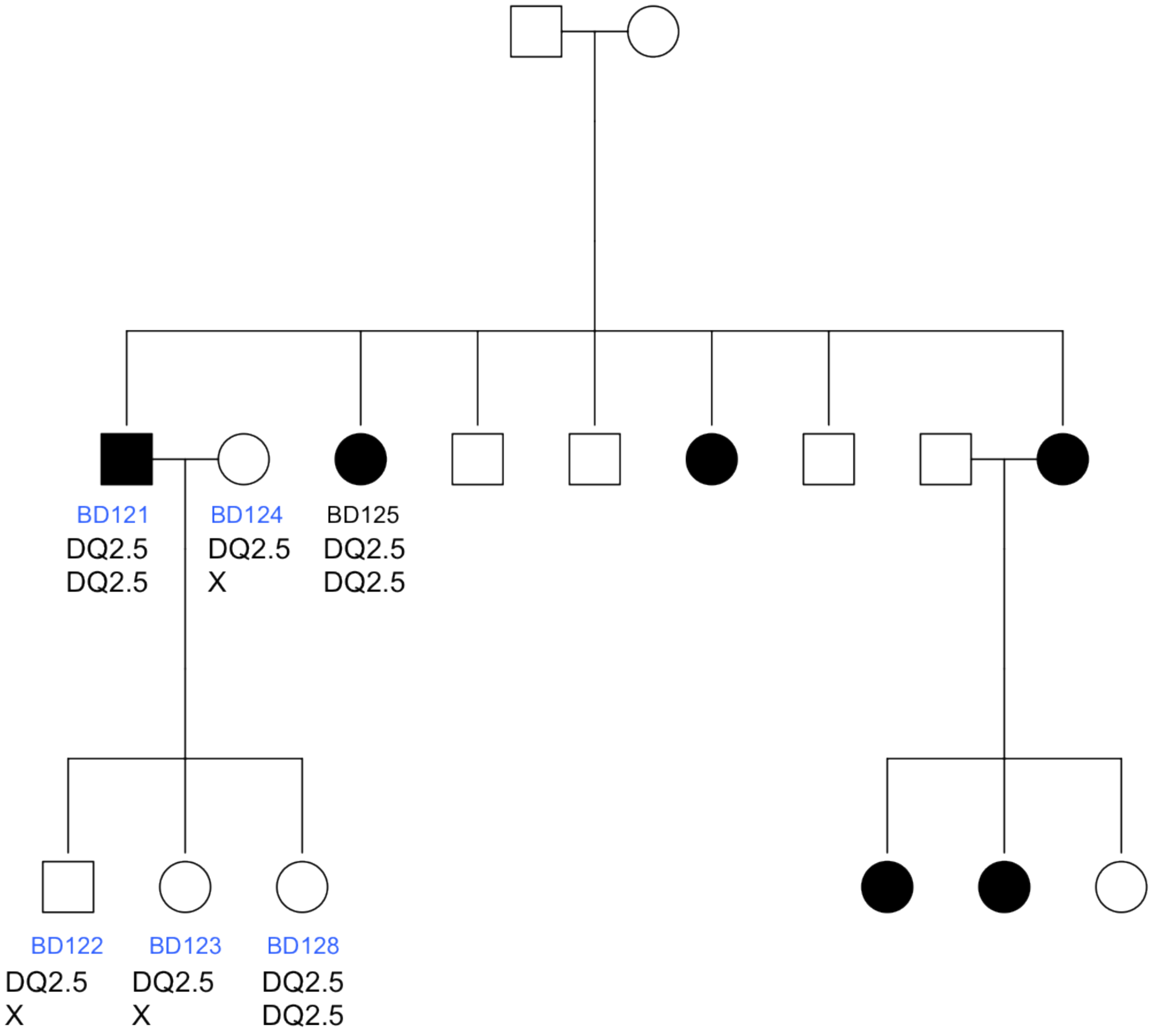

Family BR

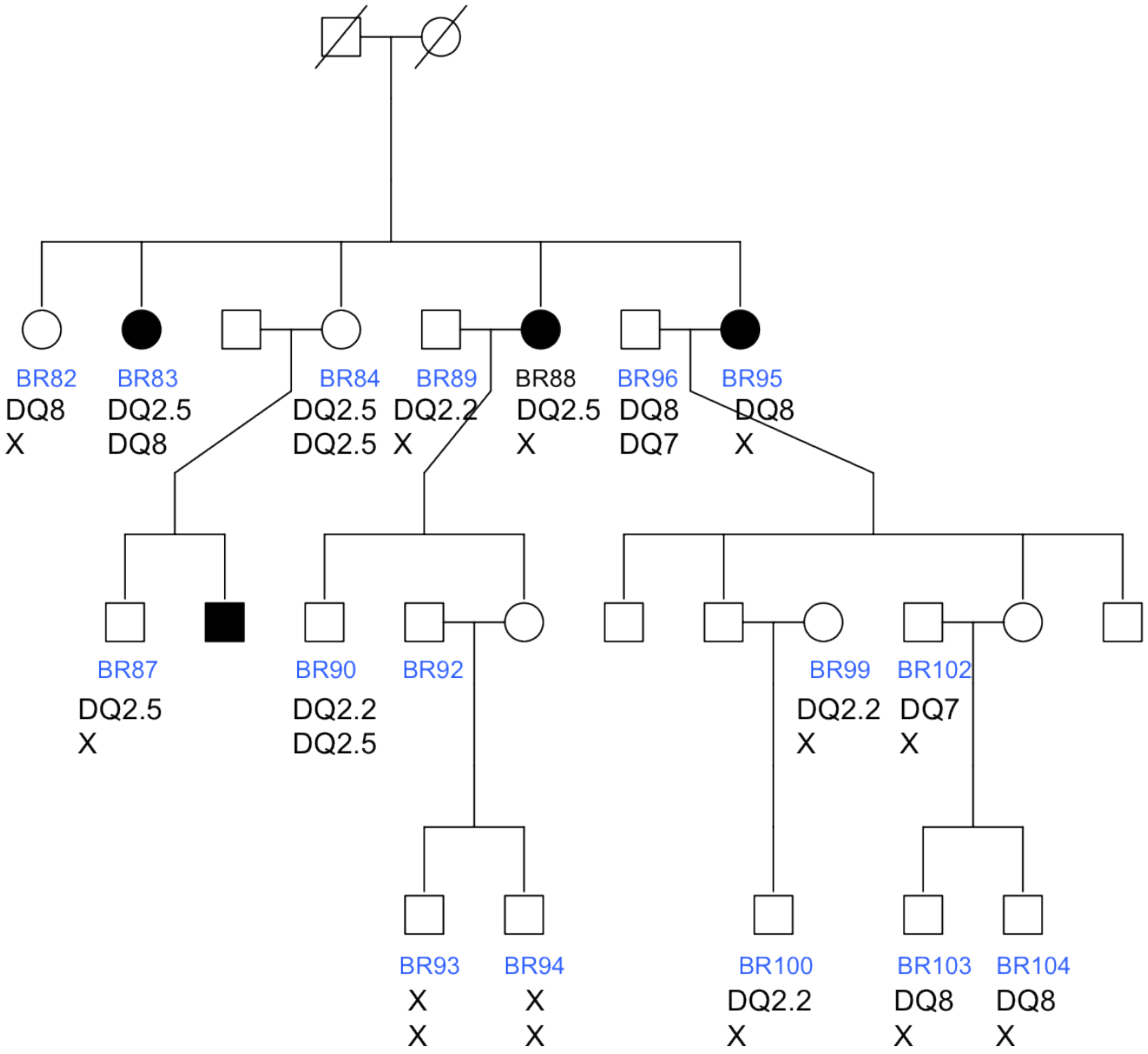
BR82
DQ8
X
BR83
DQ2.5
DQ8
BR84
DQ2.5
DQ2.5
BR89
DQ2.2
X
BR88
DQ2.5
X
BR96
DQ8
DQ7
BR95
DQ8
X
BR87
DQ2.5
X
BR90
DQ2.2
DQ2.5
BR92
BR99
DQ2.2
X
BR102
DQ7
X
BR93
X
X
BR94
X
X
BR100
DQ2.2
X
BR103
DQ8
X
BR104
DQ8
X

Family BRE

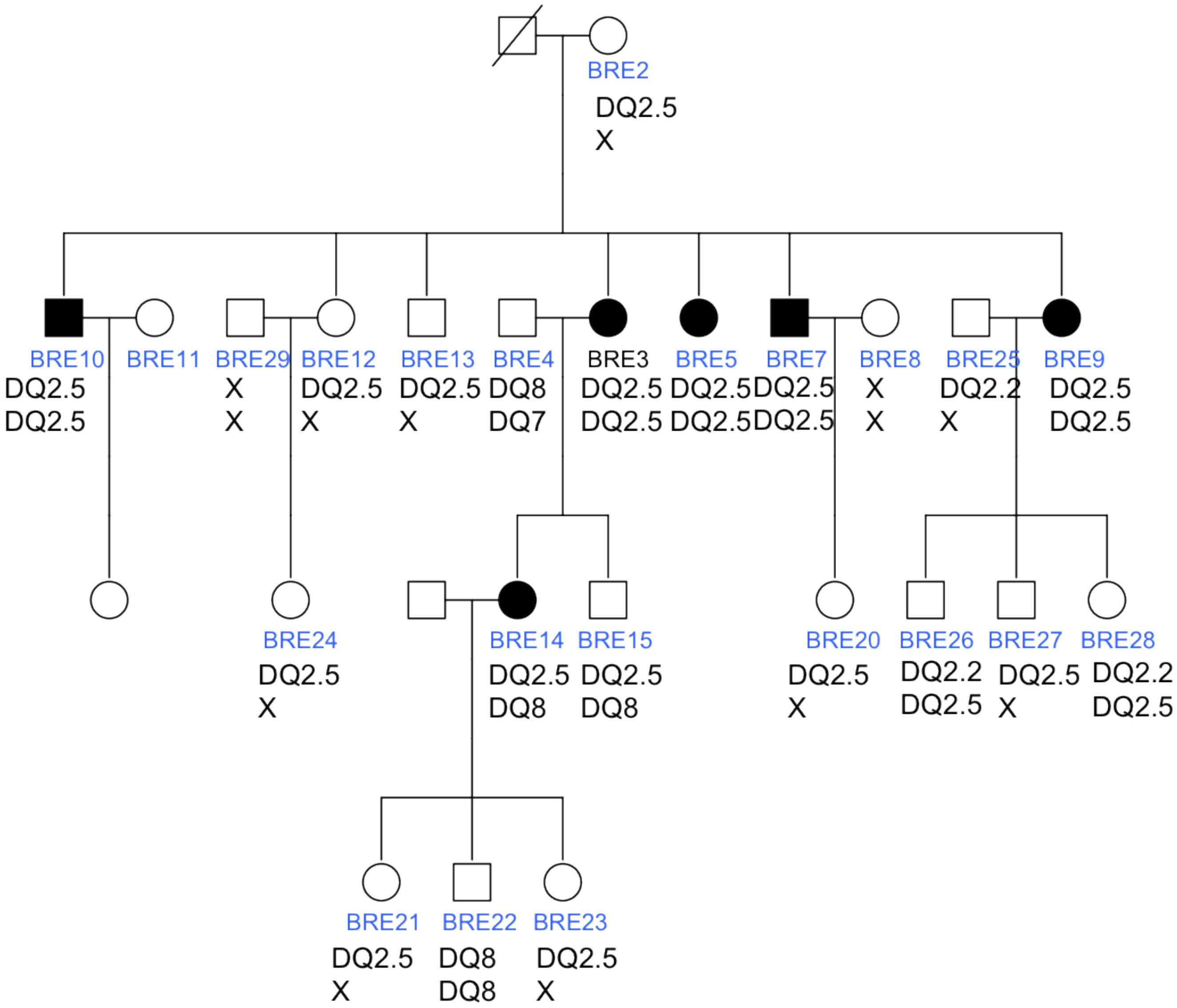
BRE2
DQ2.5
X
BRE10
DQ2.5
DQ2.5
BRE11
BRE29
X
X
BRE12
DQ2.5
X
BRE13
DQ2.5
X
BRE4
DQ8
DQ7
BRE3
DQ2.5
DQ2.5
BRE5
DQ2.5
DQ2.5
BRE7
DQ2.5
DQ2.5
BRE8
X
X
BRE25
DQ2.2
X
BRE9
DQ2.5
DQ2.5
BRE24
DQ2.5
X
BRE14
DQ2.5
DQ8
BRE15
DQ2.5
DQ8
BRE20
DQ2.5
X
BRE26
DQ2.2
DQ2.5
BRE27
DQ2.5
X
BRE28
DQ2.2
DQ2.5
BRE21
DQ2.5
X
BRE22
DQ8
DQ8
BRE23
DQ2.5
X

Family BRK

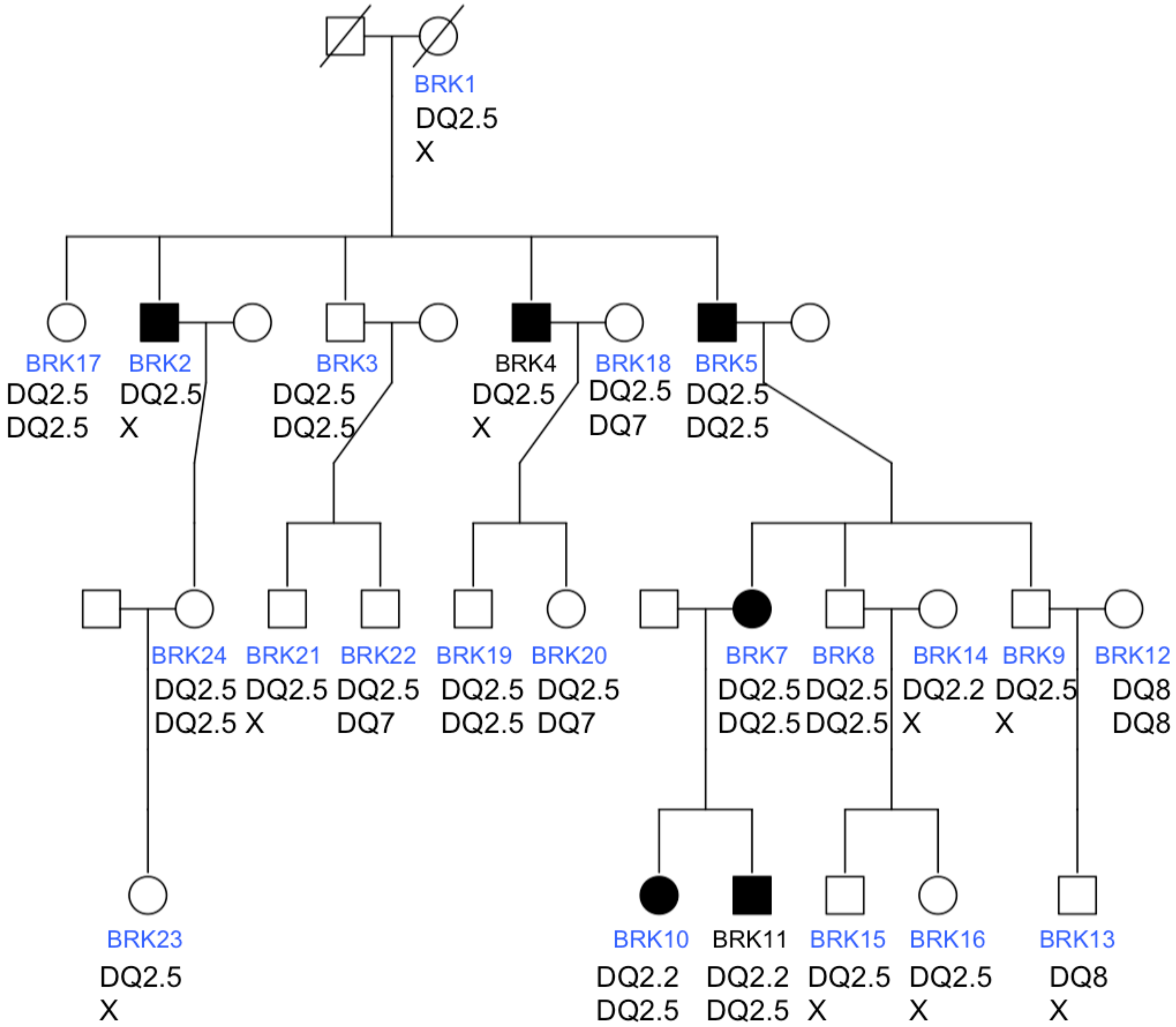
BRK1
DQ2.5
X
BRK17
DQ2.5
DQ2.5
BRK2
DQ2.5
X
BRK3
DQ2.5
DQ2.5
BRK4
DQ2.5
X
BRK18
DQ2.5
DQ7
BRK5
DQ2.5
DQ2.5
BRK24
DQ2.5
DQ2.5
BRK21
DQ2.5
X
BRK22
DQ2.5
DQ7
BRK19
DQ2.5
DQ2.5
BRK20
DQ2.5
DQ7
BRK7
DQ2.5
DQ2.5
BRK8
DQ2.5
DQ2.5
BRK14
DQ2.2
X
BRK9
DQ2.5
X
BRK12
DQ8
DQ8
BRK23
DQ2.5
X
BRK10
DQ2.2
DQ2.5
BRK11
DQ2.2
DQ2.5
BRK15
DQ2.5
X
BRK16
DQ2.5
X
BRK13
DQ8
X

Family BUT

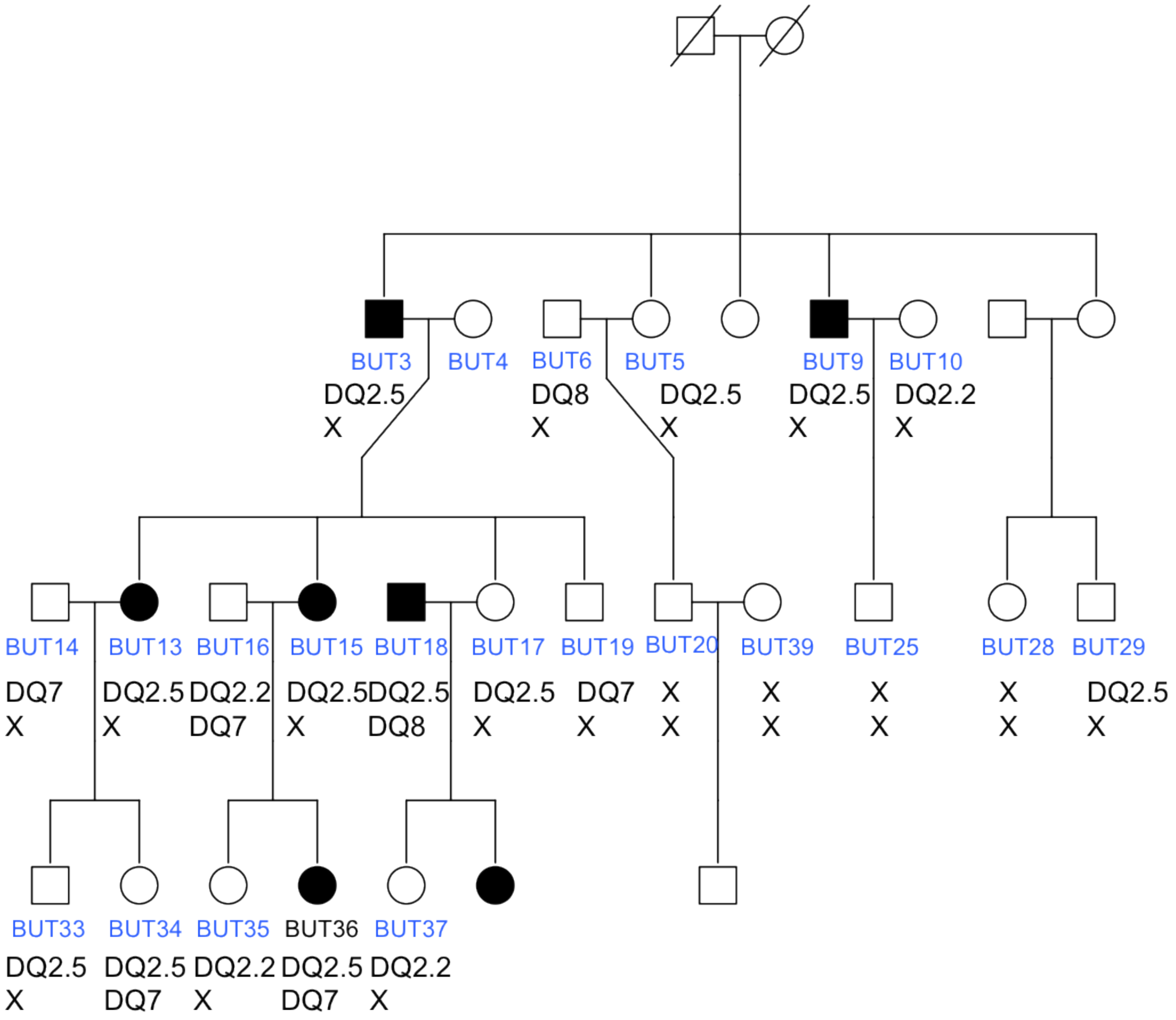
BUT3
DQ2.5
X
BUT4
BUT6
DQ8
X
BUT5
DQ2.5
X
BUT9
DQ2.5
X
BUT10
DQ2.2
X
BUT14
DQ7
X
BUT13
DQ2.5
X
BUT16
DQ2.2
DQ7
BUT15
DQ2.5
X
BUT18
DQ2.5
DQ8
BUT17
DQ2.5
X
BUT19
DQ7
X
BUT20
X
X
BUT39
X
X
BUT25
X
X
BUT28
X
X
BUT29
DQ2.5
X
BUT33
DQ2.5
X
BUT34
DQ2.5
DQ7
BUT35
DQ2.2
X
BUT36
DQ2.5
DQ7
BUT37
DQ2.2
X

Family DA

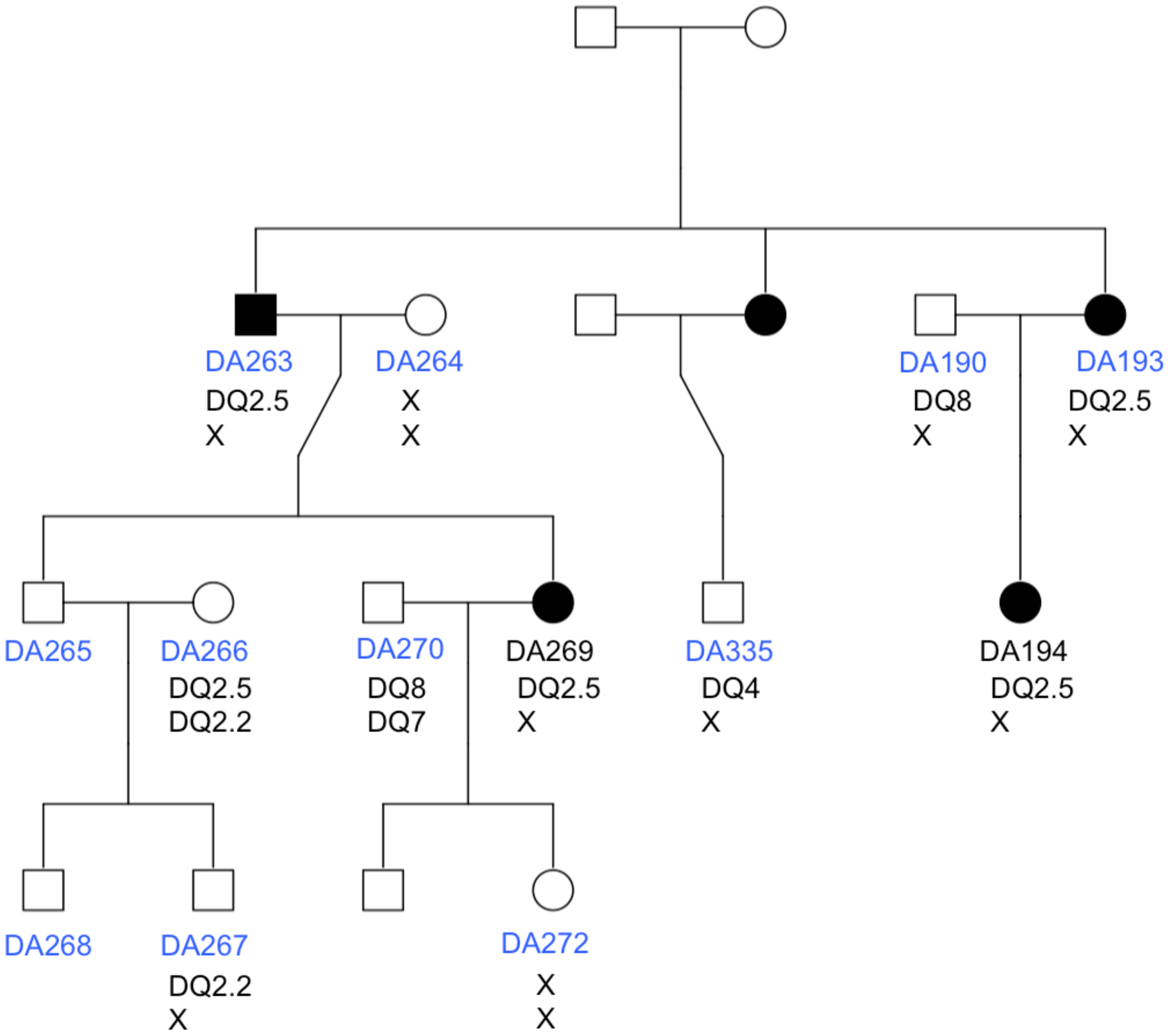

DA263
DQ2.5
X
DA264
X
X
DA190
DQ8
X
DA193
DQ2.5
X
DA265
DA266
DQ2.5
DQ2.2
DA270
DQ8
DQ7
DA269
DQ2.5
X
DA335
DQ4
X
DA194
DQ2.5
X
DA268
DA267
DQ2.2
X
DA272
X
X

Family 008

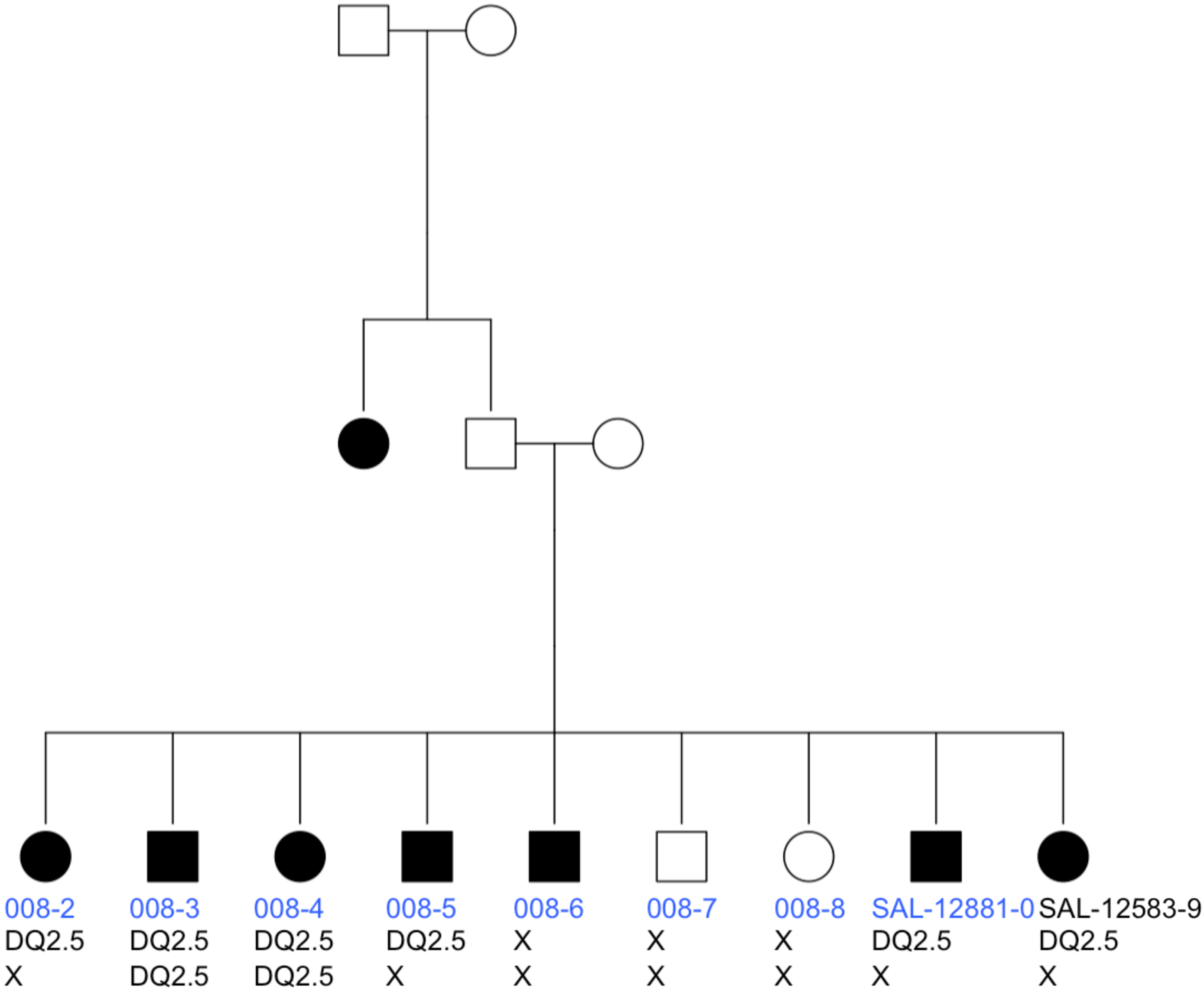

008-2
DQ2.5
X
008-3
DQ2.5
DQ2.5
008-4
DQ2.5
DQ2.5
008-5
DQ2.5
X
008-6
X
X
008-7
X
X
008-8
X
X
SAL-12881-0
DQ2.5
X
SAL-12583-9
DQ2.5
X

Family 014

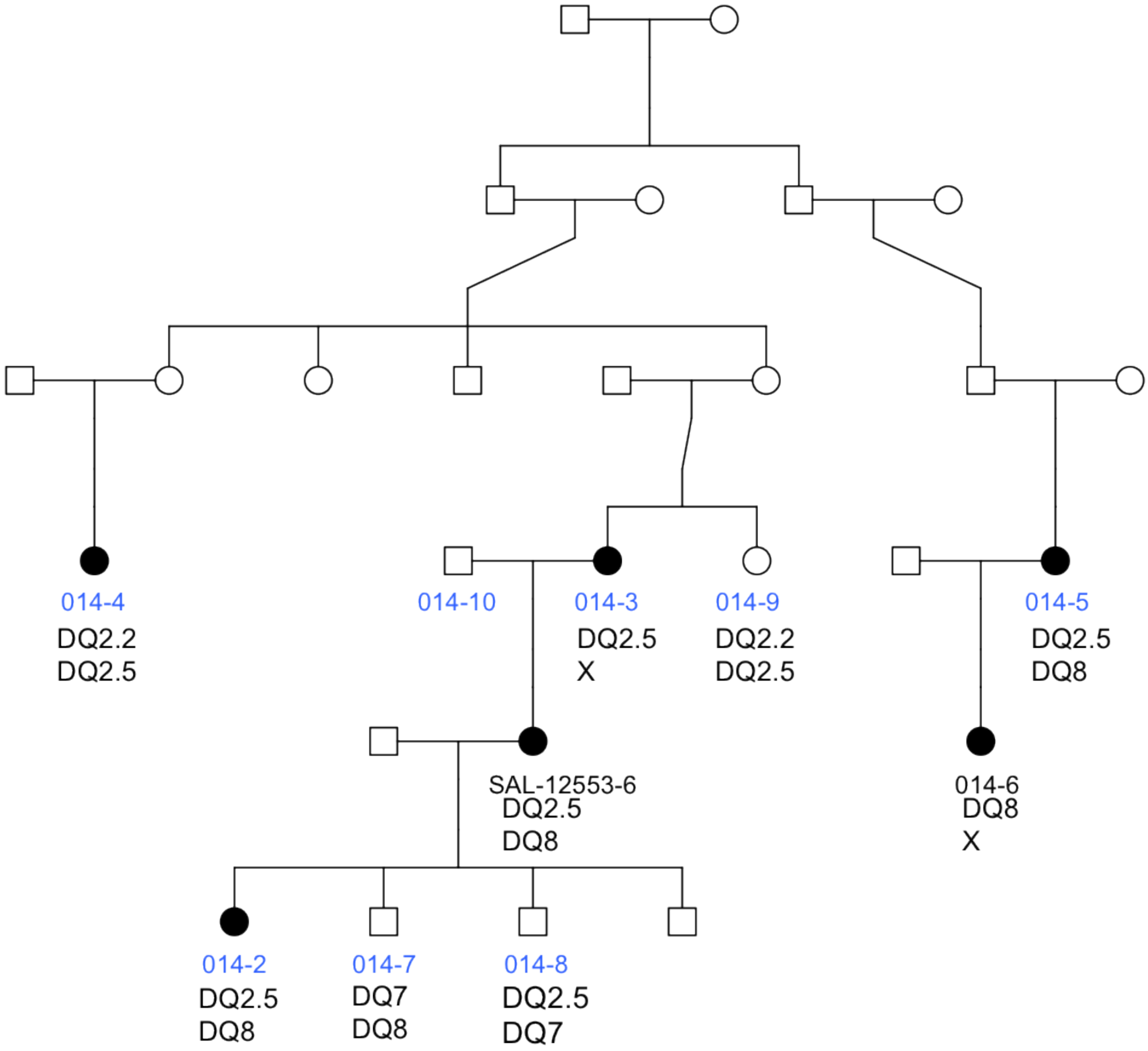
014-4
DQ2.2
DQ2.5
014-10
014-3
DQ2.5
X
014-9
DQ2.2
DQ2.5
014-5
DQ2.5
DQ8
SAL-12553-6
DQ2.5
DQ8
014-6
DQ8
X
014-2
DQ2.5
DQ8
014-7
DQ7
DQ8
014-8
DQ2.5
DQ7

Family 063

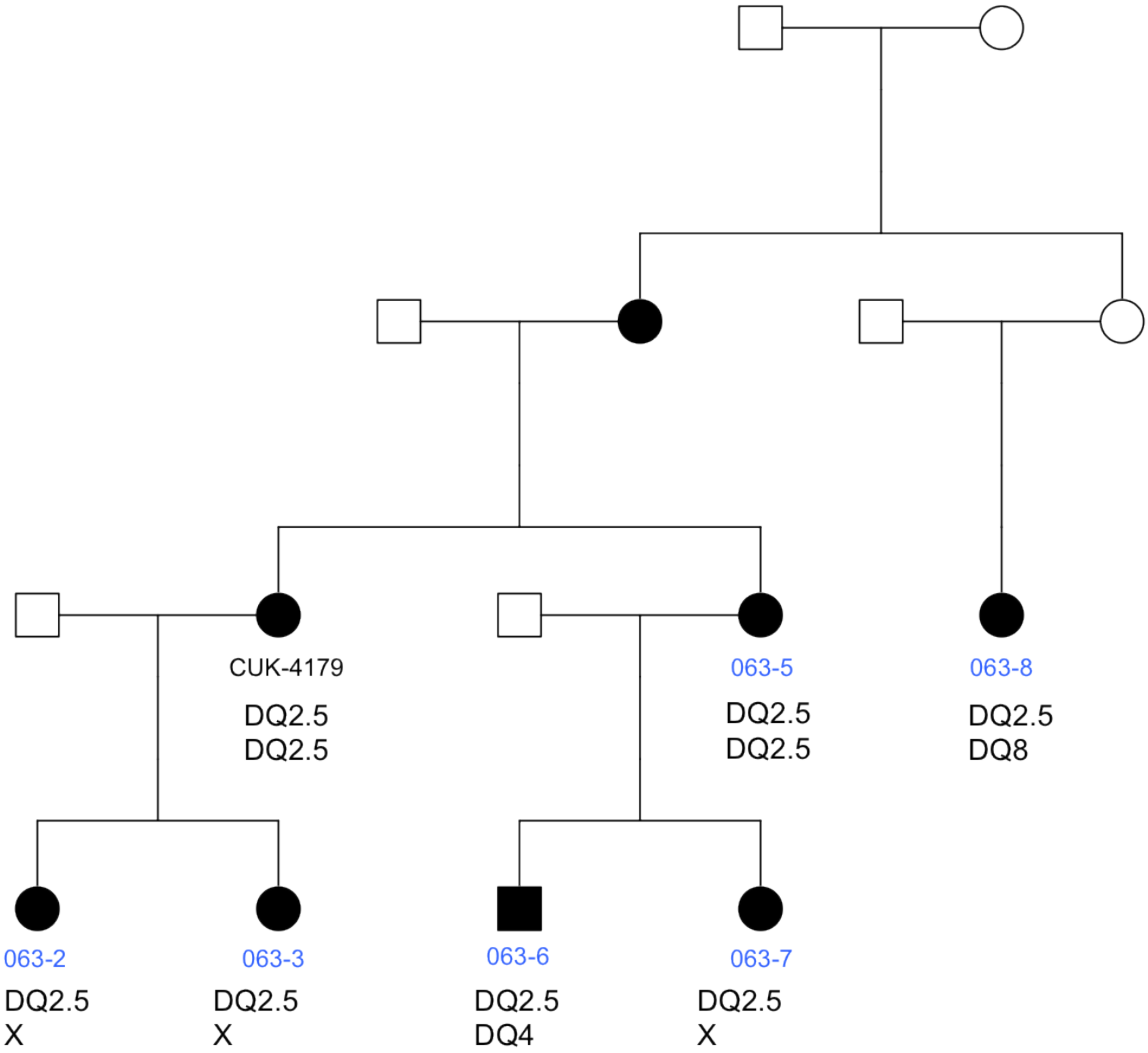

CUK-4179
DQ2.5
DQ2.5
063-5
DQ2.5
DQ2.5
063-8
DQ2.5
DQ8
063-2
DQ2.5
X
063-3
DQ2.5
X
063-6
DQ2.5
DQ4
063-7
DQ2.5
X

Family H

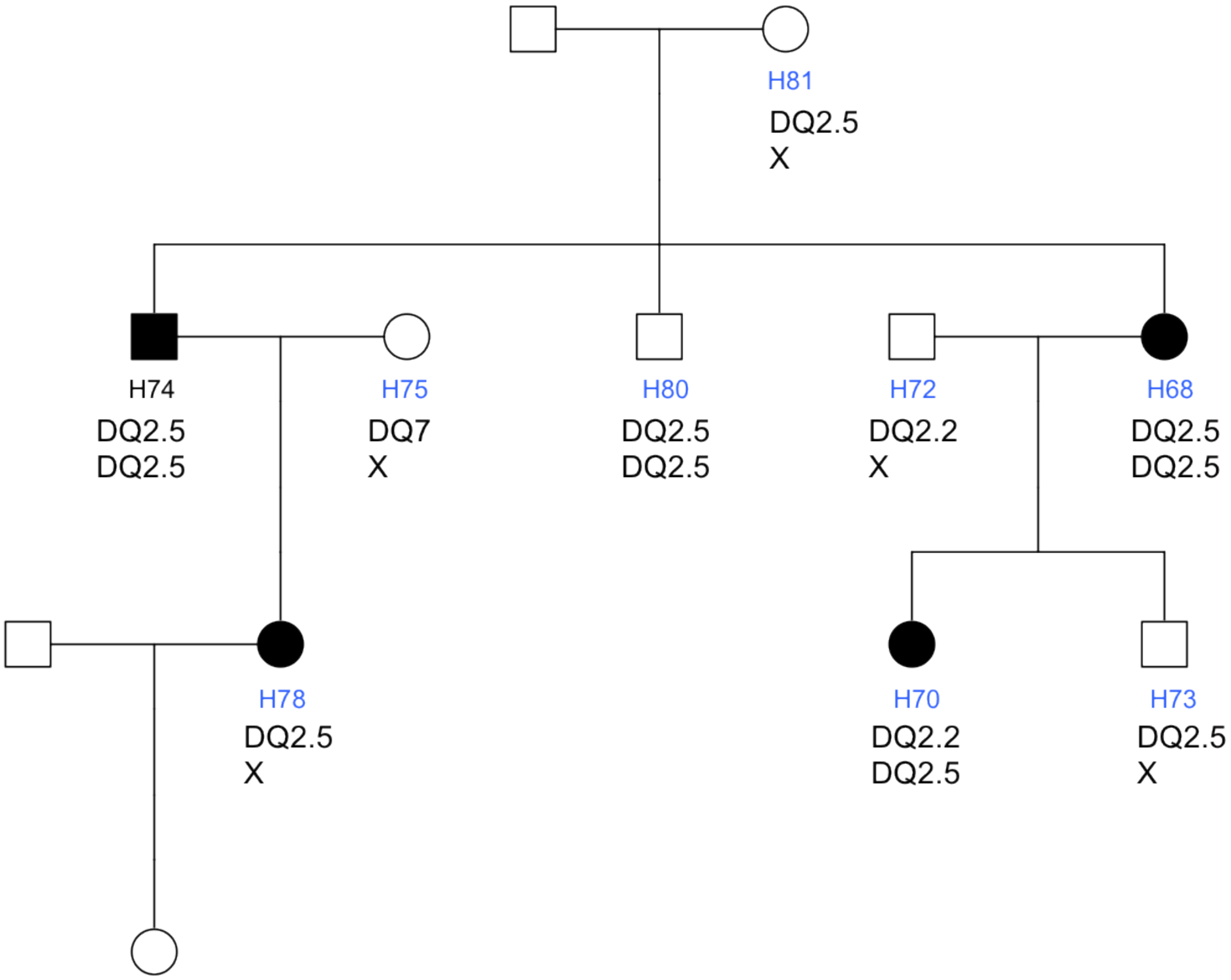
H81
DQ2.5
X
H74
DQ2.5
DQ2.5
H75
DQ7
X
H80
DQ2.5
DQ2.5
H72
DQ2.2
X
H68
DQ2.5
DQ2.5
H78
DQ2.5
X
H70
DQ2.2
DQ2.5
H73
DQ2.5
X

Family HMN

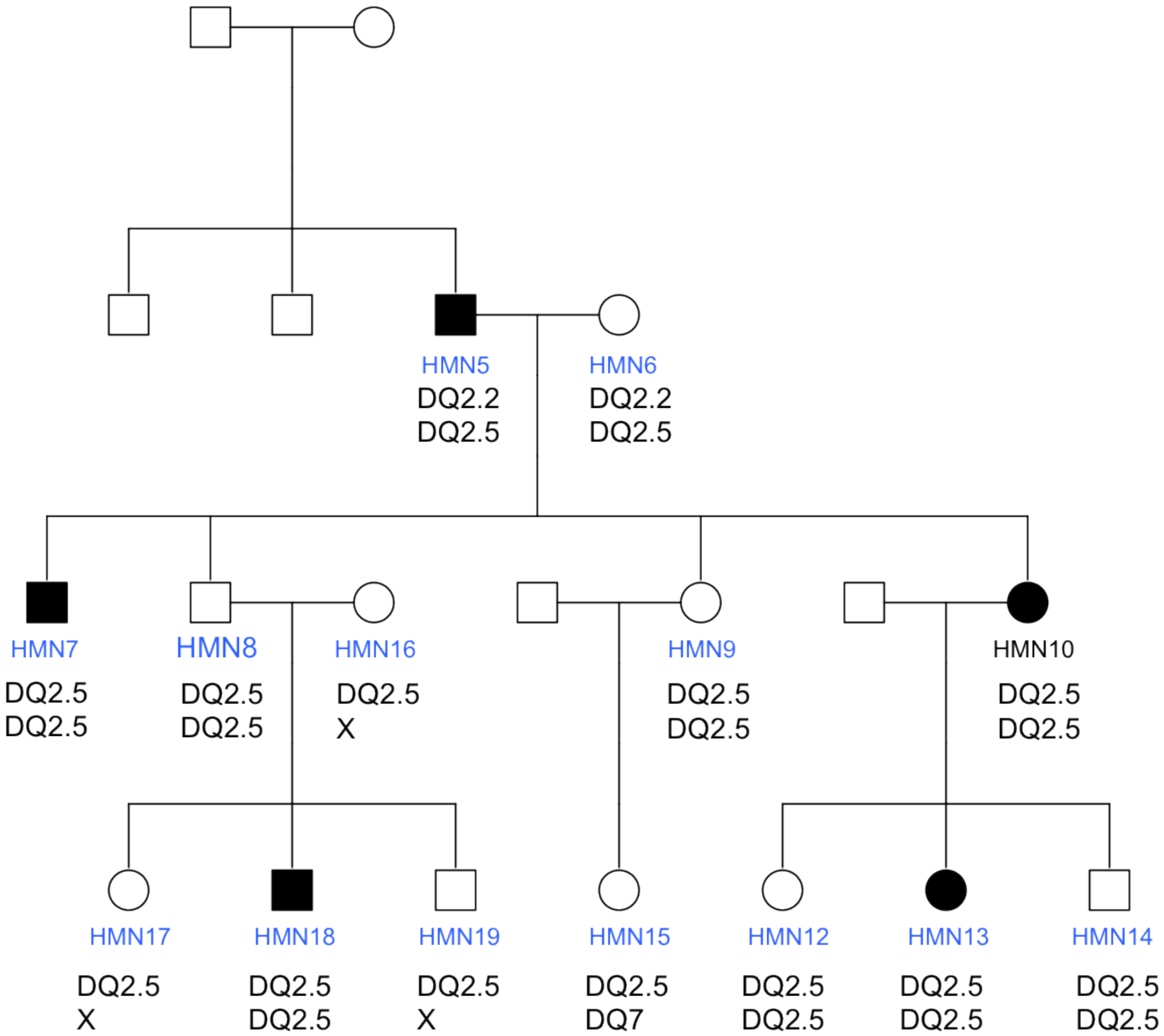
HMN5
DQ2.2
DQ2.5
HMN6
DQ2.2
DQ2.5
HMN7
DQ2.5
DQ2.5
HMN8
DQ2.5
DQ2.5
HMN16
DQ2.5
X
HMN9
DQ2.5
DQ2.5
HMN10
DQ2.5
DQ2.5
HMN17
DQ2.5
X
HMN18
DQ2.5
DQ2.5
HMN19
DQ2.5
X
HMN15
DQ2.5
DQ7
HMN12
DQ2.5
DQ2.5
HMN13
DQ2.5
DQ2.5
HMN14
DQ2.5
DQ2.5

Family SDY

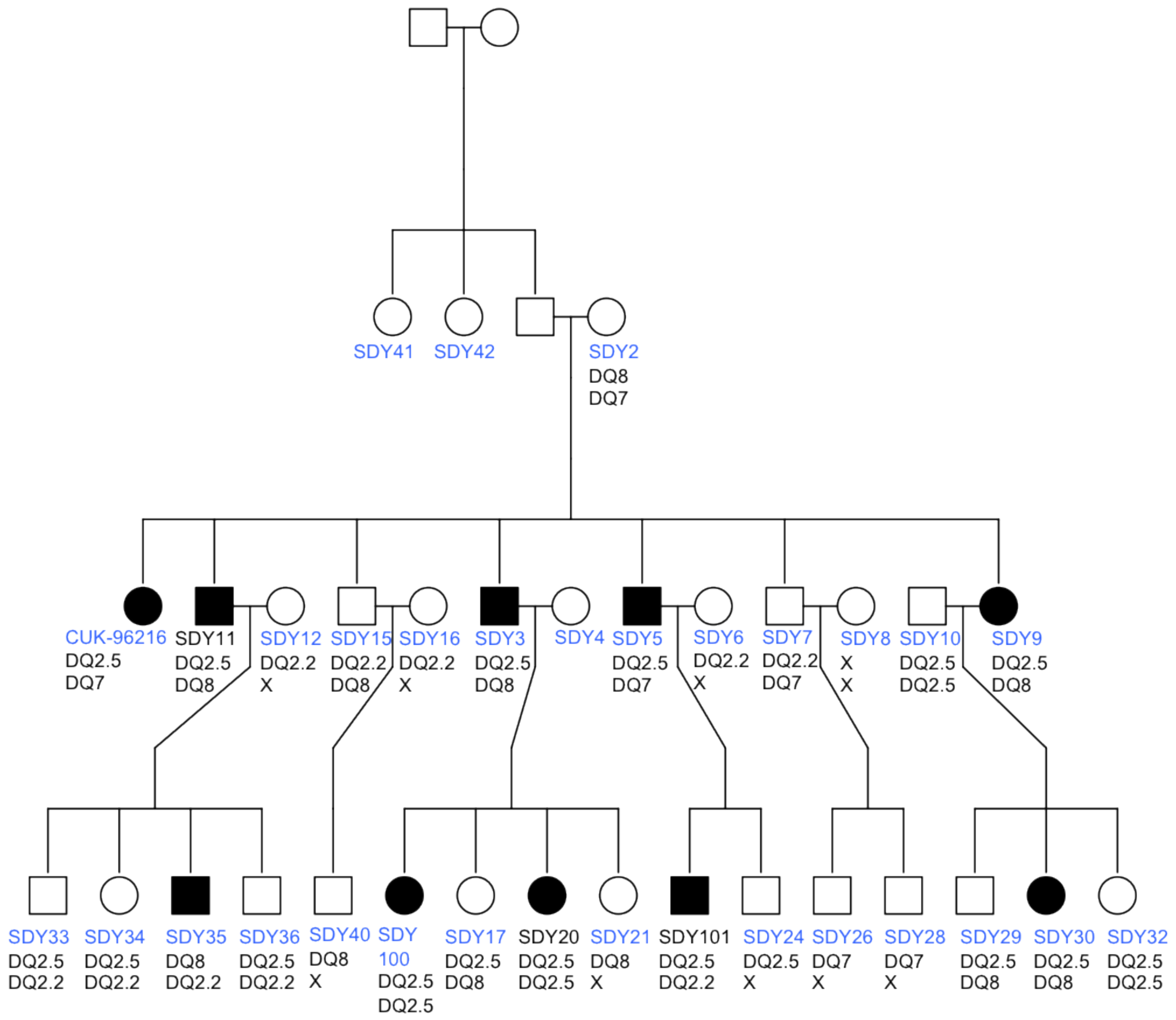

All subjects were genotyped on the Illumina ImmunoChip array. Sample names in black were exome sequenced. Sample names in blue were included in the linkage test. HLA genotypes are shown below the sample name. X denotes 'other genotype'.
