# Supplementary material for: Exome Sequencing of 75 Individuals from Multiply Affected Coeliac Families and Large Scale Resequencing Follow Up
Source: PLoS One. 2015 Jan 30;10(1):e0116845. doi: 10.1371/journal.pone.0116845 (PMC4312029; doi:10.1371/journal.pone.0116845)
Supplement: S5 Fig — A. Power calculation for single variant association testing in the discovery exome sequencing dataset for 41 exome cases (one per multiply affected family) and 220 controls. B. Power calculation for single variant association testing in 2,248 cases and 2,230 controls included in the deep amplicon resequencing study. Three scenarios are shown reflecting different allele frequencies in controls: 2% (blue line), 1% (red line) and 0.5% (black line). Y-axis shows power to detect association at p<10−4 as a function of the odds ratio parameter. (PDF) [file pone.0116845.s005.pdf]

Figure S5. Power calculations for the exome discovery and candidate gene deep amplicon resequencing studies

A. Power calculation for single variant association testing in the discovery exome sequencing dataset for 41 exome cases (one per multiply affected family) and 220 controls.

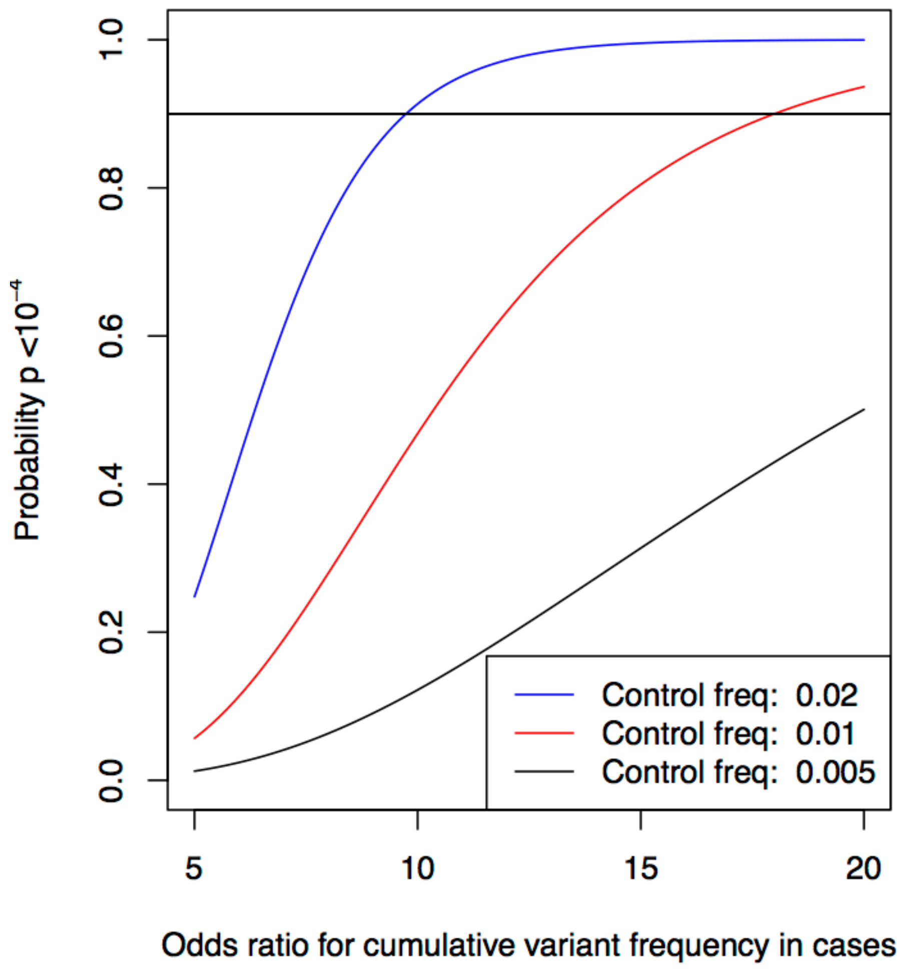

B. Power calculation for single variant association testing in 2,248 cases and 2,230 controls included in the deep amplicon resequencing study

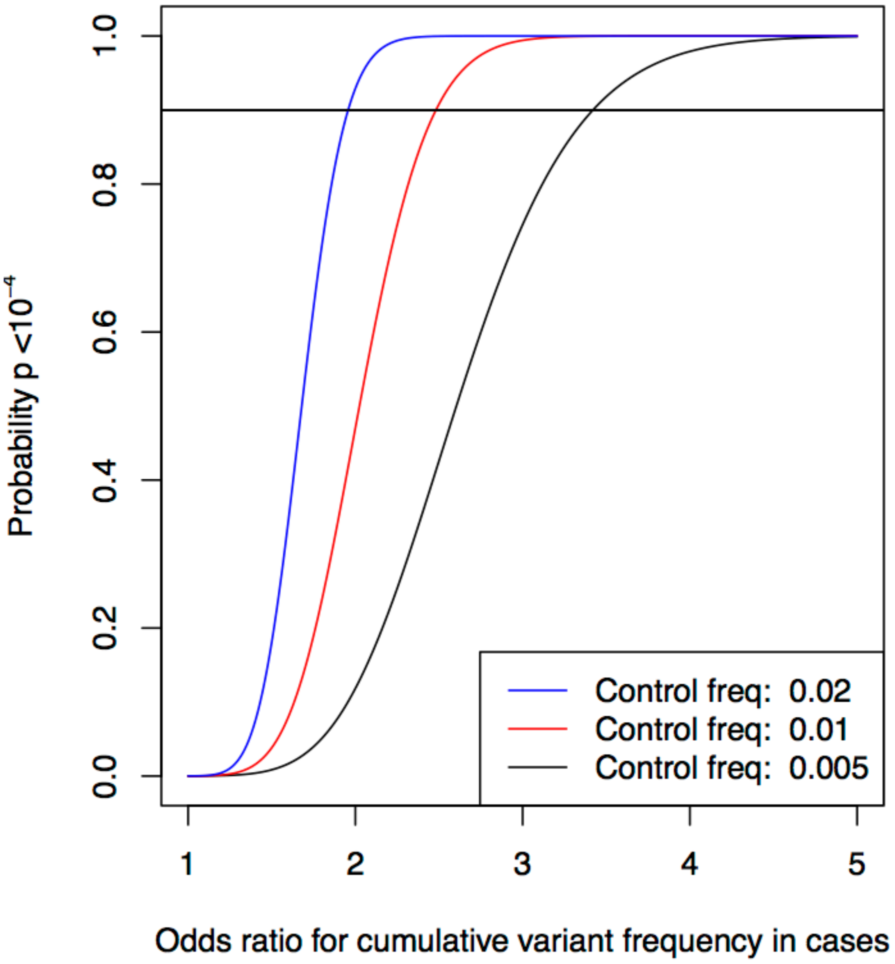

Three scenarios are shown reflecting different allele frequencies in controls: 2% (blue line), 1% (red line) and 0.5% (black line). Y-axis shows power to detect association at p<10-4 as a function of the odds ratio parameter.
