# Supplementary material for: Exome Sequencing of 75 Individuals from Multiply Affected Coeliac Families and Large Scale Resequencing Follow Up
Source: PLoS One. 2015 Jan 30;10(1):e0116845. doi: 10.1371/journal.pone.0116845 (PMC4312029; doi:10.1371/journal.pone.0116845)
Supplement: S1 Table — The numbers of affected, unaffected and status unknown individuals out of the total number of individuals is shown per family, separated by slashes. A ‘-‘ denotes unknown number of individuals. *Pedigrees selected for non-parametric linkage analysis. (DOCX) [file pone.0116845.s006.docx]

**Table S1. Total numbers of affected cases per family and numbers of sequenced coeliac cases per family.**

| **Family ID** | **Affected individuals/ Unaffected individuals/Status unknown/Total number of individuals in family** | **Number of affected individuals sequenced** | **Ethnicity** |
| --- | --- | --- | --- |
| NEU4768 | 14/65/0/79 | 2 | Denmark/UK |
| NEU4801 | 9/50/0/59 | 3 | Denmark/UK/Sweden |
| NEU7017 | 7/42/0/49 | 3 | Denmark/UK |
| NEU7058 | 7/18/0/25 | 2 | UK |
| NEU4735 | 7/16/0/23 | 3 | Denmark/Norway |
| NAL108 | 8/5/0/13 | 2 | Sweden |
| DA* | 5/13/0/18 | 2 | UK |
| BRK* | 6/19/4/29 | 2 | UK |
| BRE* | 6/19/0/25 | 1 | UK |
| HMN* | 5/15/0/25 | 1 | UK |
| BD* | 6/10/0/16 | 1 | UK |
| BR* | 4/18/4/26 | 1 | UK |
| BUT* | 7/22/1/30 | 1 | UK |
| B | 4/9/2/15 | - | UK |
| H* | 4/8/0/12 | 1 | UK |
| FAM001 | 4/-/-/- | 3 | UK |
| FAM002 | 4/-/-/- | 2 | UK |
| SDY* | 13/18/0/31 | 3 | UK |
| FAM004 | 3/-/-/- | 1 | UK |
| FAM005 | 6/-/-/- | 1 | UK |
| FAM006 | 4/-/-/- | 2 | UK |
| FAM007 | 3/-/-/- | 2 | UK |
| FAM008* | 8/-/-/- | 1 | UK |
| FAM009 | 5/-/-/- | 1 | UK |
| FAM010 | 7/-/-/- | 2 | UK |
| FAM011 | 4/-/-/- | 1 | UK |
| FAM012 | 4/-/-/- | 1 | UK |
| FAM013 | 3/-/-/- | 1 | UK |
| FAM014* | 6/6/16/28 | 2 | UK |
| FAM015 | 3/-/-/- | 1 | UK |
| FAM016 | 3/-/-/- | 1 | UK |
| FAM017 | 3/-/-/- | 1 | UK |
| FAM018 | 4/-/-/- | 1 | UK |
| FAM019 | 4/-/-/- | 1 | UK |
| FAM020 | 5/-/-/- | 1 | UK |
| FAM021 | 5/-/-/- | 1 | UK |
| FAM023 | 5/-/-/- | 1 | UK |
| FAM024 | 7/-/-/- | 1 | UK |
| FAM025 | 5/-/-/- | 1 | UK |
| FAM026 | 4/-/-/- | 1 | UK |
| FAM027 | 3/-/-/- | 1 | UK |
| FAM028 | 5/-/-/- | 1 | UK |
| FAM031 | 2/-/-/- | 1 | UK |
| FAM033 | 3/-/-/- | 1 | UK |
| FAM034 | 3/-/-/- | 1 | UK |
| FAM035 | 3/-/-/- | 1 | UK |
| FAM036 | 2/-/-/- | 1 | UK |
| FAM037 | 3/-/-/- | 1 | UK |
| FAM038 | 3/-/-/- | 1 | UK |
| FAM039 | 4/-/-/- | 1 | UK |
| FAM043 | 3/-/-/- | 1 | UK |
| FAM050 | 2/-/-/- | 1 | UK |
| FAM062 | 9/-/-/- | 1 | UK |
| FAM063* | 8/2/5/15 | 1 | UK |
| FAM065 | 5/-/-/- | 1 | UK |
| FAM066 | 1/-/-/- | 1 | UK |
| Total | 279/355/75/475 | 75 |  |

The numbers of affected, unaffected and status unknown individuals out of the total number of individuals is shown per family, separated by slashes. A‘-‘ denotes unknown number of individuals. *Pedigrees selected for non-parametric linkage analysis.
