# Supplementary material for: Exome Sequencing of 75 Individuals from Multiply Affected Coeliac Families and Large Scale Resequencing Follow Up
Source: PLoS One. 2015 Jan 30;10(1):e0116845. doi: 10.1371/journal.pone.0116845 (PMC4312029; doi:10.1371/journal.pone.0116845)
Supplement: S2 Table — The highest ImmunoChip-associated variant for CeD was selected per locus. The allele frequency of the risk allele (AF) in controls and the odds ratio (OR) for CeD, as reported by [6] are shown. Position refers to NCBI build 37. ^ Variant as reported in the CeD ImmunoChip study [6]. (DOCX) [file pone.0116845.s007.docx]

**Table S2. 57 coeliac associated variants examined in the Mangrove analysis.**

| **Chr** | **Position** | **Variant^^^** | **Risk allele** | **OR** | **AF** |
| --- | --- | --- | --- | --- | --- |
| 1 | 2529260 | imm_1_2529260 | T | 1.151 | 0.6445 |
| 1 | 25162321 | imm_1_25162321 | G | 1.059 | 0.4999 |
| 1 | 170947654 | imm_1_170947654 | T | 1.212 | 0.8145 |
| 1 | 171131275 | imm_1_171131275 | T | 1.124 | 0.8238 |
| 1 | 190779182 | imm_1_190779182 | A | 1.249 | 0.02073 |
| 1 | 190808095 | imm_1_190808095 | G | 1.308 | 0.8196 |
| 1 | 199148015 | imm_1_199148015 | C | 1.12 | 0.6844 |
| 2 | 61040333 | imm_2_61040333 | G | 1.161 | 0.3764 |
| 2 | 68499064 | imm_2_68499064 | T | 1.073 | 0.7373 |
| 2 | 102453202 | imm_2_102453202 | A | 1.202 | 0.2192 |
| 2 | 181716045 | imm_2_181716045 | C | 1.211 | 0.4172 |
| 2 | 191621279 | imm_2_191621279 | A | 1.227 | 0.9463 |
| 2 | 191656882 | imm_2_191656882 | G | 1.125 | 0.8885 |
| 2 | 191681808 | imm_2_191681808 | G | 1.08 | 0.3014 |
| 2 | 204168206 | imm_2_204168206 | T | 1.113 | 0.04242 |
| 2 | 204318641 | imm_2_204318641 | C | 1.169 | 0.2253 |
| 2 | 204478299 | imm_2_204478299 | A | 1.111 | 0.7829 |
| 3 | 33012725 | imm_3_33012725 | C | 1.121 | 0.2942 |
| 3 | 46180690 | imm_3_46180690 | C | 1.115 | 0.3606 |
| 3 | 46353029 | imm_3_46353029 | C | 1.198 | 0.3055 |
| 3 | 46459287 | imm_3_46459287 | A | 1.315 | 0.07065 |
| 3 | 120605968 | imm_3_120605968 | A | 1.126 | 0.3969 |
| 3 | 161106253 | imm_3_161106253 | C | 1.189 | 0.7082 |
| 3 | 161120372 | imm_3_161120372 | A | 1.347 | 0.118 |
| 3 | 161157622 | imm_3_161157622 | T | 1.187 | 0.4606 |
| 3 | 189602595 | imm_3_189602595 | A | 1.339 | 0.518 |
| 4 | 123257745 | imm_4_123257745 | A | 1.181 | 0.07307 |
| 4 | 123770564 | imm_4_123770564 | A | 1.412 | 0.8249 |
| 6 | 341321 | imm_6_341321 | C | 1.138 | 0.779 |
| 6 | 353079 | imm_6_353079 | C | 1.126 | 0.4672 |
| 6 | 32605884 | rs2187668 | A | 6.583 | 0.1419 |
| 6 | 90866360 | imm_6_90866360 | C | 1.106 | 0.3838 |
| 6 | 128335255 | imm_6_128335255 | T | 1.207 | 0.2412 |
| 6 | 128335748 | imm_6_128335748 | C | 1.196 | 0.8447 |
| 6 | 138043754 | imm_6_138043754 | G | 1.213 | 0.8201 |
| 6 | 138047208 | imm_6_138047208 | G | 1.288 | 0.2235 |
| 6 | 159389562 | imm_6_159389562 | A | 1.167 | 0.4367 |
| 6 | 159418255 | imm_6_159418255 | C | 1.255 | 0.07566 |
| 7 | 37384979 | 1kg_7_37384979 | G | 1.214 | 0.1058 |
| 8 | 129333242 | imm_8_129333242 | A | 1.08 | 0.7552 |
| 10 | 6430198 | imm_10_6430198 | C | 1.121 | 0.7776 |
| 10 | 80728033 | imm_10_80728033 | A | 1.147 | 0.5183 |
| 11 | 111196858 | rs7104791 | T | 1.179 | 0.2072 |
| 11 | 118579865 | rs10892258 | G | 1.179 | 0.7655 |
| 11 | 127897147 | imm_11_127897147 | T | 1.178 | 0.2083 |
| 12 | 110368991 | imm_12_110368991 | T | 1.199 | 0.4863 |
| 14 | 68329255 | imm_14_68329255 | C | 1.134 | 0.2144 |
| 15 | 75096443 | rs1378938 | A | 1.11 | 0.2683 |
| 16 | 10964118 | rs6498114 | G | 1.138 | 0.2439 |
| 16 | 11268703 | imm_16_11268703 | A | 1.102 | 0.7069 |
| 16 | 11281298 | imm_16_11281298 | A | 1.686 | 0.003385 |
| 16 | 11292457 | imm_16_11292457 | G | 1.037 | 0.1532 |
| 18 | 12833137 | imm_18_12833137 | C | 1.16 | 0.1591 |
| 18 | 12847758 | ccc-18-12847758-G-A | A | 1.216 | 0.03868 |
| 21 | 42728136 | imm_21_42728136 | A | 1.157 | 0.6976 |
| 21 | 44453549 | imm_21_44453549 | A | 1.088 | 0.8175 |
| 22 | 20309289 | imm_22_20309289 | C | 1.178 | 0.1835 |

The highest ImmunoChip-associated variant for CeD was selected per locus. The allele frequency of the risk allele (AF) in controls and the odds ratio (OR) for CeD, as reported by [6] are shown. Position refers to NCBI build 37. ^^^ Variant as reported in the CeD ImmunoChip study [6].
