# Supplementary material for: Exome Sequencing of 75 Individuals from Multiply Affected Coeliac Families and Large Scale Resequencing Follow Up
Source: PLoS One. 2015 Jan 30;10(1):e0116845. doi: 10.1371/journal.pone.0116845 (PMC4312029; doi:10.1371/journal.pone.0116845)
Supplement: S3 Table — One sample per line. *Samples sequenced twice due to initial poor capture and/or sequencing run. (DOCX) [file pone.0116845.s008.docx]

**Table S3. Summary statistics for 75 CeD familial exomes.**

| **Mean coverage** | **Number of SNVs** | **Total reads** | **Total unique reads** | **% unique reads** | **% reads on target** |
| --- | --- | --- | --- | --- | --- |
| 49.9 | 15314 | 21806499 | 17522198 | 80.4 | 85.8 |
| 38.7 | 14792 | 19488200 | 13920438 | 71.4 | 83.3 |
| 38.0 | 14319 | 19383798 | 13464140 | 69.5 | 83.2 |
| 74.3 | 15748 | 35961107 | 27931526 | 77.7 | 84.6 |
| 54.3 | 15105 | 22269704 | 20809469 | 93.4 | 88.5 |
| 51.4 | 15352 | 21261310 | 19875402 | 93.5 | 87.2 |
| 58.2 | 15210 | 25508789 | 23768557 | 93.2 | 85.0 |
| 65.7 | 15019 | 27373757 | 24610549 | 89.9 | 88.4 |
| 45.9 | 14779 | 19367470 | 18213413 | 94 | 87.1 |
| 55.9 | 14913 | 24654132 | 22656262 | 91.9 | 85.4 |
| 45.1 | 12791 | 20487543 | 19492259 | 95.1 | 86.2 |
| 60.6 | 15061 | 26787695 | 25029622 | 93.4 | 85.1 |
| 48.4 | 16663 | 20644436 | 19199835 | 93 | 84.5 |
| 107.1* | 15326 | 52997717 | 42201911 | 79.6 | 87.5 |
| 67.3 | 15519 | 28230050 | 24629384 | 87.2 | 89.4 |
| 71.4 | 14840 | 29951044 | 28034390 | 93.6 | 88.2 |
| 112.7* | 15290 | 53114021 | 43554049 | 82 | 89.3 |
| 54.9 | 14888 | 25088339 | 23566243 | 93.9 | 82.4 |
| 40.8 | 15044 | 16227946 | 14934907 | 92 | 86.6 |
| 61.1 | 15345 | 26223092 | 24003151 | 91.5 | 86.9 |
| 69.5 | 15235 | 30987209 | 28714566 | 92.7 | 86.6 |
| 67.3 | 15152 | 30396359 | 28247009 | 92.9 | 85.0 |
| 65.0 | 15192 | 29112035 | 25688348 | 88.2 | 87.0 |
| 59.8 | 15241 | 26061548 | 24223058 | 92.9 | 85.5 |
| 60.2 | 15371 | 26001856 | 23893175 | 91.9 | 86.6 |
| 60.5 | 15614 | 26383621 | 23538963 | 89.2 | 87.5 |
| 63.1 | 15259 | 34913754 | 28483442 | 81.6 | 75.0 |
| 69.2 | 15620 | 35028306 | 30299232 | 86.5 | 77.8 |
| 44.8 | 13676 | 22960721 | 15888421 | 69.2 | 84.5 |
| 51.3 | 14313 | 22526005 | 17645066 | 78.3 | 86.3 |
| 48.7 | 16638 | 48765253 | 35895980 | 73.6 | 47.3 |
| 40.2 | 16361 | 43749347 | 29222387 | 66.8 | 47.6 |
| 16.9 | 16505 | 9466873 | 7583305 | 80.1 | 79.7 |
| 13.7 | 14263 | 8611430 | 6271787 | 72.8 | 68.7 |
| 63.4 | 15612 | 25999072 | 23174204 | 89.1 | 88.2 |
| 59.1 | 15493 | 24549860 | 22278612 | 90.7 | 87.2 |
| 67.6 | 15228 | 26056990 | 24589360 | 94.4 | 86.3 |
| 34.3 | 14893 | 19743950 | 18813543 | 95.3 | 59.9 |
| 42.8 | 16164 | 35145165 | 29530782 | 84 | 51.2 |
| 42.8 | 15180 | 25898998 | 24320220 | 93.9 | 59.5 |
| 53.0 | 15280 | 20576500 | 19278197 | 93.7 | 86.2 |
| 69.8 | 15240 | 28163868 | 25607734 | 90.9 | 88.3 |
| 58.3 | 15653 | 24132862 | 21050052 | 87.2 | 85.9 |
| 64.9 | 15216 | 26144218 | 23136401 | 88.5 | 87.7 |
| 48.4 | 15425 | 21832717 | 19595612 | 89.8 | 78.4 |
| 46.9 | 15214 | 17929953 | 15887718 | 88.6 | 87.4 |
| 61.8 | 15408 | 24234228 | 21989897 | 90.7 | 87.2 |
| 51.1 | 15105 | 19669432 | 18508536 | 94.1 | 85.4 |
| 59.6 | 15201 | 22335030 | 20901435 | 93.6 | 88.8 |
| 66.4 | 15250 | 26369954 | 25009023 | 94.8 | 87.0 |
| 92.0* | 15160 | 41779561 | 35245721 | 84.4 | 90.6 |
| 22.2 | 12770 | 59120951 | 15719553 | 26.6 | 51.5 |
| 72.9 | 14038 | 37348622 | 31995783 | 85.7 | 85.6 |
| 41.8 | 12884 | 19913810 | 17546341 | 88.1 | 85.2 |
| 59.7 | 14655 | 25179001 | 23652659 | 93.9 | 84.6 |
| 58.6 | 14921 | 30653765 | 28810630 | 94 | 73.3 |
| 40.4 | 12460 | 19672961 | 17778083 | 90.4 | 80.2 |
| 66.5 | 14611 | 27731440 | 25907859 | 93.4 | 83.9 |
| 52.6 | 16087 | 30728026 | 28824424 | 93.8 | 63.3 |
| 102.7* | 14018 | 51141008 | 38837379 | 75.9 | 89.8 |
| 37.9 | 12103 | 17947394 | 16605739 | 92.5 | 85.4 |
| 84.0* | 14795 | 51658474 | 42532697 | 82.3 | 75.0 |
| 28.8 | 14310 | 11087549 | 10610810 | 95.7 | 87.6 |
| 36.4 | 12811 | 15527983 | 14628854 | 94.2 | 90.3 |
| 35.0 | 13038 | 17239755 | 14750871 | 85.6 | 85.1 |
| 33.1 | 12302 | 14658611 | 13469866 | 91.9 | 86.8 |
| 63.6 | 15078 | 54726041 | 41168886 | 75.2 | 61.4 |
| 38.3 | 13337 | 18527186 | 17142783 | 92.5 | 77.9 |
| 34.9 | 12223 | 14623026 | 13531857 | 92.5 | 87.5 |
| 33.8 | 12631 | 14677702 | 13580414 | 92.5 | 87.2 |
| 38.7 | 13100 | 16706684 | 15391261 | 92.1 | 91.1 |
| 40.2 | 13101 | 19139632 | 16817288 | 87.9 | 83.4 |
| 47.7 | 15437 | 19426782 | 17179335 | 88.4 | 89.4 |
| 60.9 | 14931 | 24104415 | 21420247 | 88.9 | 87.9 |

One sample per line. *Samples sequenced twice due to initial poor capture and/or sequencing run.
