# Supplementary material for: Exome Sequencing of 75 Individuals from Multiply Affected Coeliac Families and Large Scale Resequencing Follow Up
Source: PLoS One. 2015 Jan 30;10(1):e0116845. doi: 10.1371/journal.pone.0116845 (PMC4312029; doi:10.1371/journal.pone.0116845)
Supplement: S4 Table — *Information taken from Ensemble genome Browser, release 71. (DOCX) [file pone.0116845.s009.docx]

**Table S4. Candidate genes for deep amplicon resequencing selected from exome data of 75 CeD individuals from multiply affected families.**

| **Gene** | **Analysis selected from:** | **cDNA size*** | **Exons*** |
| --- | --- | --- | --- |
| *ACOT8* | Linkage | 1,168bp | 6 |
| *ARHGAP25* | Linkage | 2,979bp | 11 |
| *C1QBP* | Shared in familial exomes | 1,169bp | 6 |
| *CD180* | Case Control/Shared | 2,726bp | 3 |
| *CD1C* | Case Control | 1,435bp | 6 |
| *CERK* | Case Control | 4,450bp | 13 |
| *CRLF3* | Case Control | 2,917bp | 8 |
| *EBI3* | Case Control | 1,128bp | 5 |
| *EPAS1* | Linkage | 5,160bp | 16 |
| *GRM4* | Linkage | 3,879bp | 10 |
| *HAS1* | Shared in familial exomes | 2,087bp | 5 |
| *IFNW1* | Case Control | 1,514bp | 1 |
| *IKZF3* | Case Control | 9,667bp | 8 |
| *IL12RB1* | Shared in familial exomes | 2,100bp | 17 |
| *KCNJ16* | Linkage | 4,002bp | 5 |
| *MALT1* | Linkage | 8,789bp | 17 |
| *MAP4K2* | Shared in familial exomes | 2,955bp | 32 |
| *NLRC4* | Linkage | 3,581bp | 10 |
| *RAF1* | Shared in familial exomes | 3,300bp | 17 |
| *TNFRSF10A* | Shared in familial exomes | 2,714bp | 10 |
| *TNFRSF13B* | Shared in familial exomes | 1,357bp | 5 |
| *TNFRSF21* | Segregation | 3,595bp | 6 |
| *TRAF4* | Shared in familial exomes | 2,921bp | 7 |
| *TULP1* | Linkage | 2,162bp | 15 |

*Information taken from Ensemble genome Browser, release 71
