# Supplementary material for: Exome Sequencing of 75 Individuals from Multiply Affected Coeliac Families and Large Scale Resequencing Follow Up
Source: PLoS One. 2015 Jan 30;10(1):e0116845. doi: 10.1371/journal.pone.0116845 (PMC4312029; doi:10.1371/journal.pone.0116845)
Supplement: S5 Table — Immunologically important genes downloaded from Gene Ontology (http://wiki.geneontology.org/index.php/Immunology) and used as a guide to select genes in immune mediated pathways for the candidate gene resequencing study. Immune functional information collected from NCBI (http://www.ncbi.nlm.nih.gov/gene/), T1Dbase (http://t1dbase.org) and Online Mendelian Inheritance in Man (OMIM; http://www.ncbi.nlm.nih.gov/omim) websites. Association with autoimmune diseases at the genome-wide level gathered from the Catalog of Published Genome Wide Association Studies (http://www.genome.gov/). (DOCX) [file pone.0116845.s010.docx]

**Table S5. Immune function and autoimmune disease associations of 24 candidate genes selected for deep amplicon resequencing.**

| **Gene** | **Known Immune Function** | **Known association (at the genome-wide association level) and/or function in other autoimmune diseases or gut related disorder** |
| --- | --- | --- |
| *ACOT8* | - | Best gene in the linkage region but no known immune function or association with an autoimmune disease. |
| *ARHGAP25* | - | Best gene the in linkage region but no known immune function or association with an autoimmune disease. |
| *C1QBP* |  | Gene is not known to be associated with any autoimmune disease. |
| *CD180* | Cell surface molecule that belongs to the toll-like receptor (TLR) family, controlling B cell recognition and lipopolysaccharide signalling by working alongside TLR4. | Gene is not known to be associated with any autoimmune disease. |
| *CD1C* | Belongs to the CD1 family of glycoproteins that are involved in the presentation of lipid and glycolipid antigens to T cells. | Gene is not known to be associated with any autoimmune disease. |
| *CERK* | An enzyme that catalyzes ATP and ceramide into ADP and ceramide-1-phosphate. | Gene is not known to be associated with any autoimmune disease. |
| *CRLF3* | Gene encodes for a cytokine receptor-like factor that is present in the neurofibromatosis type 1 tumour, therefore not specifically immune mediated. | Gene is not known to be associated with any autoimmune disease. |
| *EBI3* | The encoded gene forms a heterodimer with *IL27* and the response of this gene to Epstein-Barr virus induces B lymphocyte expression. | Gene is not known to be associated with any autoimmune disease. |
| *EPAS1* | Transcription factor that produces the hypoxia-inducible factor 2-alpha protein. | Gene is not known to be associated with any autoimmune disease. |
| *GRM4* | - | Best gene in the linkage region but no known immune function or association with any autoimmune disease. |
| *HAS1* | Hyaluronan has a variety of functions, including wound healing and tissue repair. | Gene is not genome wide associated to any autoimmune disease, however serum concentrations of hyaluronan differ in rheumatoid arthritis patients [1] and glucocorticoids, a treatment of rheumatoid arthritis, suppresses *HAS1* [2]. |
| *IFNW1* | Belongs to the interferon-omega family of genes, of which *IFNW1* is presumed to be the only functional gene. | Gene is not known to be associated with any autoimmune disease. |
| *IKZF3* | This zinc-finger protein is a transcription factor involved in the regulation of B lymphocyte proliferation and differentiation. | SNPs in this gene are genome wide associated with inflammatory bowel disease [3], primary biliary cirrhosis [4], ulcertative colitis [5], Crohn’s disease [6] and rheumatoid arthritis [7]. |
| *IL12RB1* | This receptor for interleukin 12 is essential for resistance to multiple pathogens, such as mycobacterium tuberculosis. | Gene is not known to be associated with any autoimmune disease. |
| *KCNJ16* | - | Best gene in the linkage region but no known immune function or association with any autoimmune disease. |
| *MALT1* | - | Best gene in the linkage region but no known immune function.  A SNP in this gene is associated with multiple sclerosis [8]. |
| *MAP4K2* | This serine/threonine protein kinase can be activated by TNF-alpha to activate MAP kinases. There is also differential expression in B lymphocytes. | Gene is not known to be associated with any autoimmune disease. |
| *NLRC4* | A bacterial inflammasome involved in the innate immune response. | Gene is not known to be associated with any autoimmune disease. |
| *RAF1* | This gene encodes for a MAP kinase. | Gene is not known to be associated with any autoimmune disease. |
| *TNFRSF10A* | A TNF receptor that activates tumor necrosis factor-related apoptosis and induces cell apoptosis. | Gene is not known to be associated with any autoimmune disease. |
| *TNFRSF13B* | A TNF receptor that induces activation of transcription factors and interacts with a TNF ligand. | Gene not known to be associated with any autoimmune disease, however a SNP in this gene is associated with immunoglobin G levels [9]. |
| *TNFRSF21* | A TNF receptor that induces cell apoptosis; studies in mice highlight a role in T-helper cell activation involved in inflammation [10, 11]. | Gene is not known to be associated with any autoimmune disease. |
| *TRAF4* | This gene encodes for a TNF receptor associated family protein and interacts with a neurotrophin receptor. | Gene is not known to be associated with any autoimmune disease. |
| *TULP1* | - | Best gene in the linkage region but no known immune function. A SNP in this gene is associated with hepatitis C virus induced liver fibrosis [12]. |

Immunologically important genes downloaded from Gene Ontology (http://wiki.geneontology.org/index.php/Immunology) and used as a guide to select genes in immune mediated pathways for the candidate gene resequencing study. Immune functional information collected from NCBI (http://www.ncbi.nlm.nih.gov/gene/), T1Dbase (http://t1dbase.org) and Online Mendelian Inheritance in Man (OMIM; http://www.ncbi.nlm.nih.gov/omim) websites. Association with autoimmune diseases at the genome-wide level gathered from the Catalog of Published Genome Wide Association Studies (http://www.genome.gov/).

1. Partsch G, Leeb B, Stancikova M, Raffayova H, Eberl G, et al. (1996) Low serum hyaluronan in psoriatic arthritis patients in comparison to rheumatoid arthritis patients. Clin Exp Rheumatol 14: 381-386.

2. Stuhlmeier KM and Pollaschek C. (2004) Glucocorticoids inhibit induced and non-induced mRNA accumulation of genes encoding hyaluronan synthases (HAS): hydrocortisone inhibits HAS1 activation by blocking the p38 mitogen-activated protein kinase signaling pathway. Rheumatology 43: 164-169.

3. Jostins L, Ripke S, Weersma RK, Duerr RH, McGovern DP, et al. (2012) Host-microbe interactions have shaped the genetic architecture of inflammatory bowel disease. Nature 491: 119-124.

4. Nakamura M, Nishida N, Kawashima M, Aiba Y, Tanaka A, et al. (2012) Genome-wide association study identifies TNFSF15 and POU2AF1 as susceptibility loci for primary biliary cirrhosis in the Japanese population Am J Hum Genet 91: 721-728.

5. Anderson CA, Boucher G, Lees CW, Franke A, D’Amato M, et al. (2011) Meta-analysis identifies 29 additional ulcerative colitis risk loci, increasing the number of confirmed associations to 47. Nat Genet 43: 246-256.

6. Franke A, McGovern DP, Barrett JC, Wank K, Radford-Smith GL, et al. (2010) Genome-wide meta-analysis increases to 71 the number of confirmed Crohn’s disease susceptibility loci. Nat Genet 42: 1118-1125.

7. Stahl EA, Raychaudhuri S, Remmers EF, Xie G, Eyre S, et al. (2010) Genome-wide association study meta-analysis identifies seven new rheumatoid arthritis risk loci. Nat Genet 42: 508-514

8. International Multiple Sclerosis Genetics Consortium, Wellcome Trust Case Control Consortium 2, Sawcer S, Hellenthal G, Pirinen M, et al. (2011) Genetic risk and a primary role for cell-mediated immune mechanisms in multiple sclerosis. Nature 476: 214-219.

9. Liao M, Ye F, Zhang B, Huang L, Xiao Q, et al. (2012) Genome-wide association study identifies common variants at TNFRSF13B associated with IgG level in a healthy Chinese male population. Genes Immun 13: 509-513.

10. Liu J, Na S, Glasebrook A, Fox N, Solenberg PJ, et al. (2001) Enhanced CD4+ T cell proliferation and Th2 cytokine production in DR6-deficient mice. Immunity 15: 23-34.

11. Zhao H, Yan M, Wang H, Erickson S, Grewal IS, et al. (2001) Impaired c-Jun amino terminal kinase activity and T cell differentiation in death receptor 6-deficient mice. J Exp Med 194: 1441-1448.

12. Patin E, Kutalik Z, Guergnon J, Bibert S, Nalpas B, et al. Genome-wide association study identifies variants associated with progression of liver fibrosis from HCV infection. Gastroenterology 143: 1244
